# Multimodal immunogenomic biomarker analysis of tumors from pediatric patients enrolled to a phase 1-2 study of single-agent atezolizumab

**DOI:** 10.1038/s43018-023-00534-x

**Published:** 2023-04-10

**Authors:** Arash Nabbi, Arnavaz Danesh, Osvaldo Espin-Garcia, Stephanie Pedersen, Johanna Wellum, Lingyan Helen Fu, Joseph N. Paulson, Birgit Geoerger, Lynley V. Marshall, Tanya Trippett, Gianluca Rossato, Trevor J. Pugh, Katherine E. Hutchinson

**Affiliations:** 1 Princess Margaret Cancer Centre, University Health Network, Toronto, Ontario Canada; 2 Department of Epidemiology and Biostatistics, Western University, London, Ontario Canada; 3 Dalla Lana School of Public Health and Department of Statistical Sciences, University of Toronto, Toronto, Ontario Canada; 4 Clinical Biomarker Operations, Product Development Oncology, Genentech, South San Francisco, CA USA; 5 Department of Biostatistics, Product Development, Genentech, South San Francisco, CA USA; 6 Gustave Roussy Cancer Centre, Department of Pediatric and Adolescent Oncology, INSERM U1015, Université Paris-Saclay, Villejuif, France; 7 The Royal Marsden NHS Foundation Trust and the Institute of Cancer Research, London, UK; 8 Memorial Sloan Kettering Cancer Center, New York, NY USA; 9 Product Development Clinical Oncology, F. Hoffmann-La Roche, Basel, Switzerland; 10 Department of Medical Biophysics, University of Toronto, Toronto, Ontario Canada; 11 Ontario Institute for Cancer Research, Toronto, Ontario Canada; 12 Oncology Biomarker Development, Genentech, South San Francisco, CA USA

**Keywords:** Paediatric cancer, Cancer immunotherapy, Tumour biomarkers, Cancer

## Abstract

We report herein an exploratory biomarker analysis of refractory tumors collected from pediatric patients before atezolizumab therapy (iMATRIX-atezolizumab, NCT02541604). Elevated levels of CD8^+^ T cells and PD-L1 were associated with progression-free survival and a diverse baseline infiltrating T-cell receptor repertoire was prognostic. Differential gene expression analysis revealed elevated expression of *CALCA* (preprocalcitonin) and *CCDC183* (highly expressed in testes) in patients who experienced clinical activity, suggesting that tumor neoantigens from these genes may contribute to immune response. In patients who experienced partial response or stable disease, elevated Igα2 expression correlated with T- and B-cell infiltration, suggesting that tertiary lymphoid structures existed in these patients’ tumors. Consensus gene co-expression network analysis identified core cellular pathways that may play a role in antitumor immunity. Our study uncovers features associated with response to immune-checkpoint inhibition in pediatric patients with cancer and provides biological and translational insights to guide prospective biomarker profiling in future clinical trials.

## Abstract (web summary)

Nabbi et. al. analyze immunohistochemistry, comprehensive genomic profiling, RNA-sequencing, and TCR-sequencing data from 66 pediatric patients with cancer from a phase 1–2 clinical trial (iMATRIX-atezo) to nominate biomarkers associated with response to immunotherapy in pediatric cancer.

## Main

Although immune checkpoint inhibition (ICI) elicits responses in 20–40% of adult patients with cancer^1^, early-phase clinical trials have indicated objective response in only 5–11% of pediatric patients with varying cancer types^2–4^. The KEYNOTE-051 study used programmed death ligand-1 (PD-L1) immunohistochemistry (IHC) as a biomarker to stratify patients, but this alone was not sufficient to select likely responders^3^. A comprehensive assessment of biomarkers that may influence responses to ICI in pediatric patients with cancer is lacking. To address this and to inform patient selection in future trials, we leveraged tumor samples and data (immunohistochemistry (IHC), RNA sequencing (RNA-seq), comprehensive genomic profiling (CGP), and T-cell receptor (TCR) sequencing) collected during the iMATRIX trial of atezolizumab (iMATRIX-atezo; NCT02541604)^4^ in pediatric patients with refractory cancers to execute a broad, multimodal biomarker analysis.

## Results

**Fig. 1:**
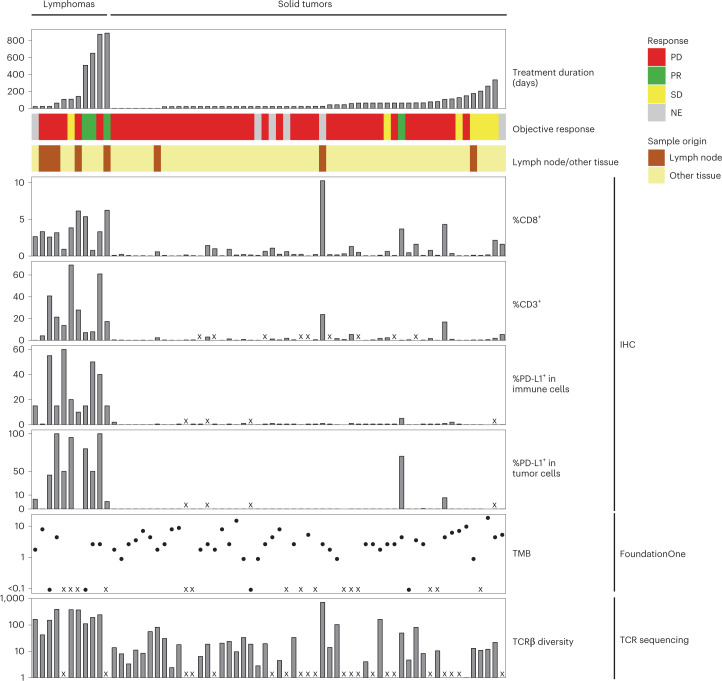
Overview of baseline sample molecular features with patient outcomes from the iMATRIX-atezo study dataset. ‘Other tissue’ indicates metastatic or primary tumors from one of the following: lung, soft tissues, bone, adrenal gland, kidney, brain or unannotated tissue types. Each bar corresponds to a single patient (*n* = 66 patients). PD, progressive disease. PR, partial response. SD, stable disease. NE, not evaluable.

Figure 1 displays the molecular characteristics of samples collected before treatment with atezolizumab under the iMATRIX-atezo trial. This cohort consisted of 66 pre-treatment samples spanning lymphomas (Hodgkin, *n* = 8 and non-Hodgkin, *n* = 3) and solid tumors (neuroblastoma (NBL; *n* = 9), osteosarcoma (OS; *n* = 8), rhabdomyosarcoma (RMS; *n* = 8), Ewing sarcoma (EWS; *n* = 6), Wilms tumor (*n* = 6), mixed sarcoma (SARC; *n* = 6), atypical teratoid rhabdoid tumors/rhabdoid tumor (ATRT/RT; *n* = 4)) or other tumor types (others; *n* = 8)) (Supplementary Table 1). Site of specimen collection consisted of lung (*n* = 11), lymph node (*n* = 11), soft tissues (*n* = 10), bone (*n* = 7), adrenal gland (*n* = 5), kidney (*n* = 5), brain (*n* = 5), and other tissue types (*n* = 12) (Supplementary Table 1). We determined the percentage of immune cells positive for CD8 and CD3 surface proteins using IHC; PD-L1 staining on tumor cell and immune cell compartments using the Ventana SP142 assay (*n* = 62); infiltrating TCRβ diversity using hybridization capture method (CapTCR-seq^5^) (*n* = 45); and genomic profiles and tumor mutational burden (TMB) from CGP with the FoundationOne (F1) next-generation sequencing assay (*n* = 51) (Supplementary Table 1).

### High levels of CD8^+^ T cells were associated with improved progression-free survival

**Fig. 2:**
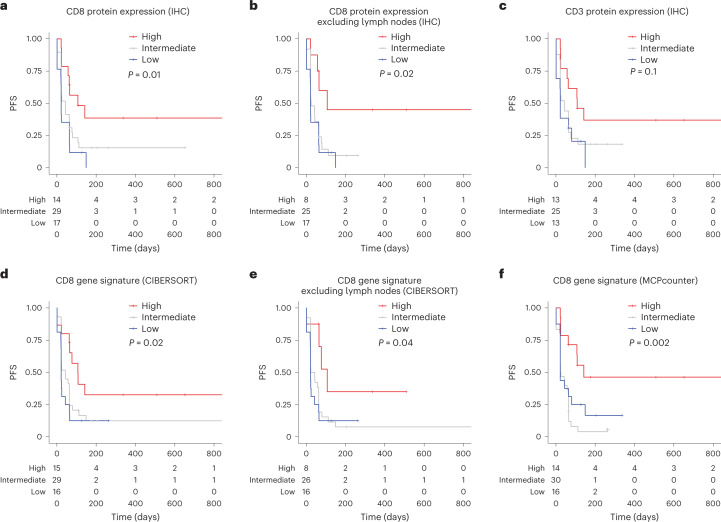
PFS on atezolizumab is associated with baseline infiltrating T-cell levels as determined by IHC and RNA-seq immune deconvolution analysis. **a**–**c**, Kaplan–Meier analysis of CD8 IHC staining in all samples (*n* = 60 patients, **a**), in samples excluding those originated from lymph nodes (*n* = 50 patients, **b**) and CD3 IHC staining (*n* = 51 patients, **c**). **d**,**e**, Kaplan–Meier analysis of CD8 gene signature obtained from CIBERSORT in all samples (*n* = 60 patients, **d**), in samples excluding those originated from lymph nodes (*n* = 50 patients, **e**) and CD8 gene signature obtained from MCPCounter (*n* = 60 patients, **f**) (high, ≥75th percentile; intermediate, 25th–75th percentile; low, ≤25th percentile) (log-rank test).

**Extended Data Fig. 1:**
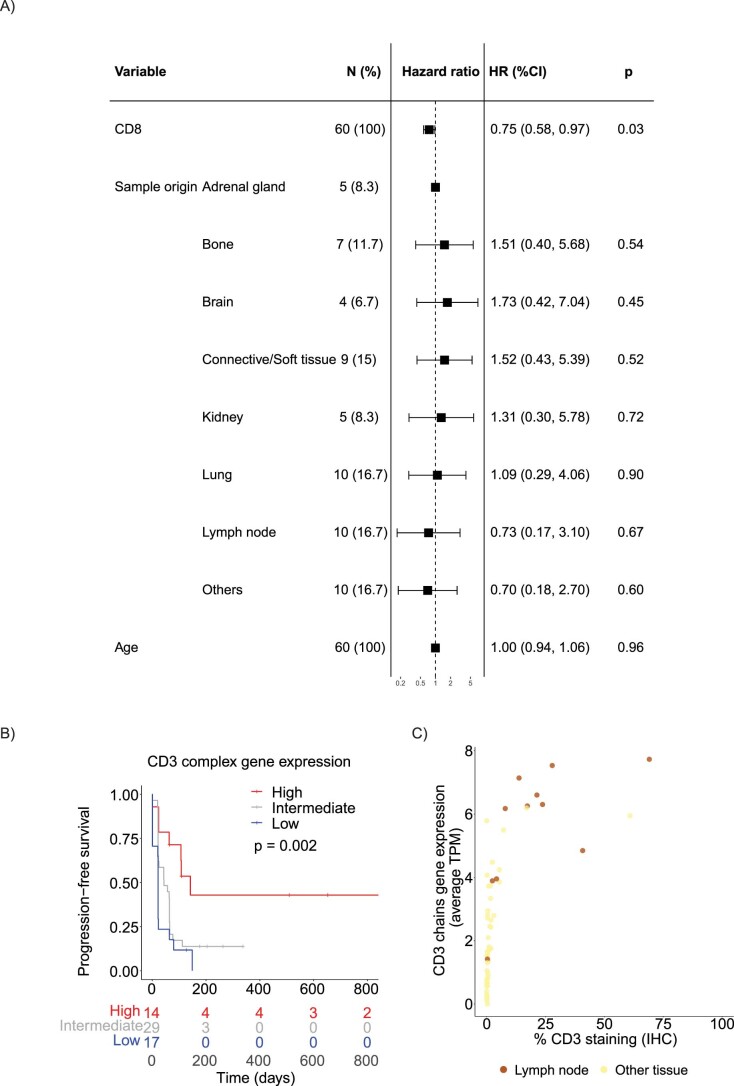
Baseline CD8 staining is associated with PFS in pediatric patients treated with atezolizumab in the iMATRIX-atezo study. (**A**) Multivariable Cox proportional hazards model for CD8 IHC staining adjusted for sample origin and age (two-sided Wald test; n = 60 biologically independent samples). Center indicates hazard ratio, and lines indicate confidence intervals. (**B**) Kaplan–Meier plot showing PFS associations of CD3 complex gene expression in the iMATRIX-atezo dataset (high, ≥75th percentile; intermediate, 25th–75th percentile; low, ≤25th percentile) (log-rank test, N = 60 patients). (**C**) Scatterplot showing correlation between CD3 chains gene expression (y-axis) and % CD3 staining by IHC (x-axis). Colors denote sample origin as lymph node (brown) or one of the following: lung, soft tissues, bone, adrenal gland, kidney, brain, or unannotated tissue types (yellow) (N = 57 samples).

We asked whether levels of T cells in tumor specimens pre-treatment were associated with progression-free survival (PFS). The percentage of cells staining positive for CD8 ranged from 0.01% to 10.3% (median 0.22%), while the percentage of CD3^+^ cells varied more widely from 0.0006% to 69.1% (median 0.57%) (Fig. 1). As expected, lymphoma samples harbored higher levels of CD8^+^ and CD3^+^ cells compared to solid tumors (median 3.3% and 17.3% for CD8 and CD3 in lymphoma versus 0.16% and 0.38% in solid tumors). Patients whose samples harbored %CD8^+^ cells in the upper quartile (≥1.24%) exhibited improved PFS compared to patients with low or intermediate %CD8^+^ staining (log-rank test, *P* = 0.01; Fig. 2a). This significant association persisted upon exclusion of lymph node samples (log-rank test, *P* = 0.02, Fig. 2b). A multivariable Cox proportional hazards model adjusting for sample origin and age confirmed improved PFS for patients with samples harboring higher %CD8^+^ cells (hazard ratio (HR) 0.7, 95% confidence interval (CI) 0.58–0.97, *P* = 0.03; Extended Data Fig. 1a); however, the overall levels of T-cell infiltration as determined by CD3 staining were not associated with PFS across the three groups (high, intermediate and low staining) in a Kaplan–Meier analysis (log-rank test, *P* = 0.1; Fig. 2c). In contrast, we found average expression of CD3 chains (*CD3D*, *CD3G* and *CD3E*) was associated with improved PFS (log-rank test, *P* = 0.002; Extended Data Fig. 1b). The correlation between CD3 protein and gene expression showed high variability, particularly in samples with <10% protein levels (Extended Data Fig. 1c). Thus, gene and protein expression are not necessarily correlated and assay sensitivity should be considered when identifying biomarkers.

**Extended Data Fig. 2:**
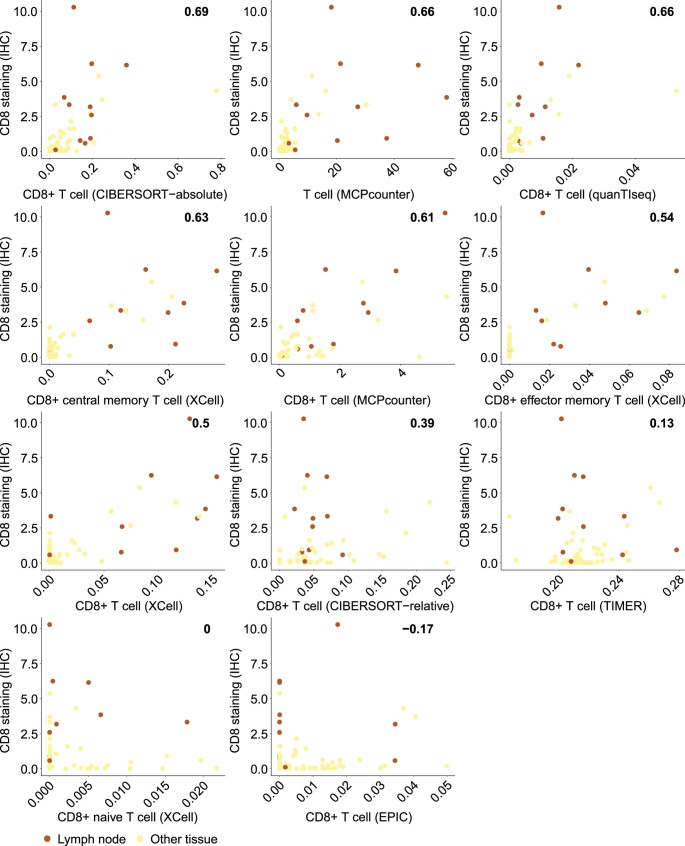
Correlations between CD8 IHC staining and RNA-seq deconvolution estimates. CD8 T-cell estimates (x-axes) were derived using six published immune deconvolution tools and compared to the percentage of CD8 staining (IHC, y-axes) from iMATRIX-atezo tumor tissue samples. Values represent the Spearman coefficients for each comparison. Colors denote sample origin as lymph node (brown) or one of the following: lung, soft tissues, bone, adrenal gland, kidney, brain, or unannotated tissue types (yellow) (N = 66 samples).

**Extended Data Fig. 3:**
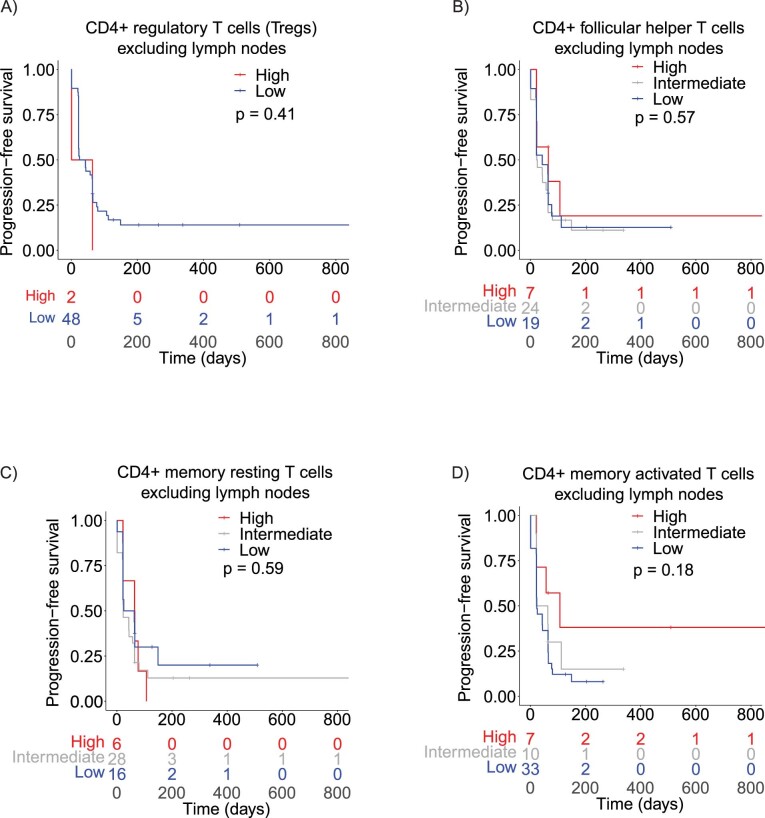
CD4+ T-cell estimates are not associated with PFS in the iMATRIX-atezo study. Kaplan–Meier analyses to determine prognostic associations of CD4+ regulatory (**A**), follicular helper (**B**), resting memory (**C**), and activated memory (**D**) T-cell estimates inferred by CIBERSORT in the iMATRIX-atezo dataset excluding samples collected from lymph nodes. Two samples with Treg estimates >0 showed no significant association with PFS (high, ≥75th percentile; intermediate, 25th–75th percentile; low, ≤25th percentile) (log-rank test, N = 50 patients).

To determine whether RNA-seq deconvolution analysis can also be used to infer levels of CD8^+^ T cells, we compared levels of estimated CD8^+^ T cells derived from six deconvolution tools^6^ with the levels of CD8 immunostaining from iMATRIX-atezo (Extended Data Fig. 2). We found the strongest correlation between RNA-seq estimates from the CIBERSORT algorithm^7^ and CD8 IHC scores (Spearman *r* = 0.69; Extended Data Fig. 2). Patients whose baseline samples exhibited CIBERSORT CD8^+^ T-cell estimates higher than the third quantile (≥0.09) had improved PFS on atezolizumab (log-rank test, *P* = 0.02; Fig. 2d). This association remained significant upon the removal of lymph node samples (log-rank test, *P* = 0.04; Fig. 2e). We observed similar associations using T-cell estimates from the MCPCounter algorithm^8^ (log-rank test, *P* = 0.002; Fig. 2f). We did not see a significant association between estimates for CD4^+^ T cells and regulatory T cells with PFS (Extended Data Fig. 3).

**Extended Data Fig. 4:**
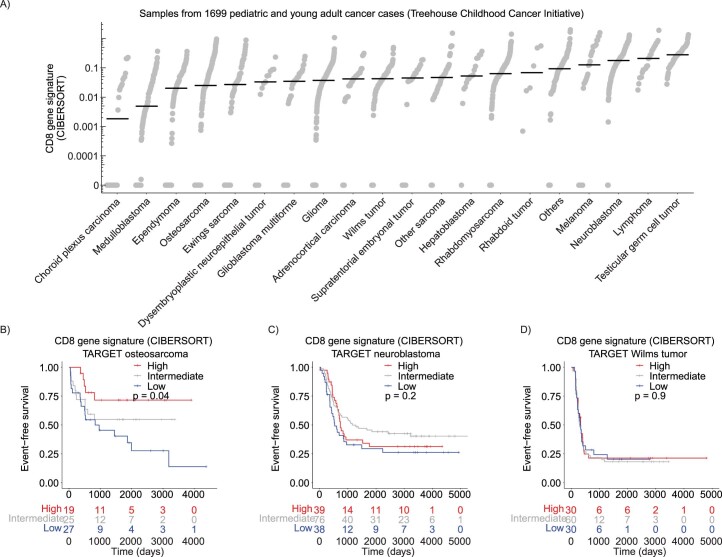
Distribution of CD8+ T-cell estimates in pediatric cancers and their associations with EFS in a non-ICI setting. (**A**) We obtained gene expression profiles of 1,699 tumor samples from children and young adults (<30 years of age) with 20 pediatric cancer types from the Treehouse Childhood Cancer Initiative and inferred levels of CD8+ T cells using gene signature from CIBERSORT (N = 1,699 patients). (**B**–**D**) Kaplan–Meier analyses to determine prognostic associations of CD8+ T-cell estimates (CIBERSORT) in non-ICI treated OS (**B**, N = 71 patients), NBL (**C**, N = 153 patients) and Wilms tumor (**D**, N = 120 patients) cohorts obtained from the NCI TARGET portal (https://ocg.cancer.gov/programs/target). Values are dichotomized using quartiles in each cohort (high, ≥75th percentile; intermediate, 25th–75th percentile; low, ≤25th percentile) (log-rank test). Source data

To place these findings in the context of a larger dataset and potentially identify additional pediatric cancer types with inherently elevated CD8 expression to which this predictive association may translate in a future ICI trial, we applied the CIBERSORT algorithm to tumor RNA-seq data from children and young adult patients with cancer (aged <30 years) from the Treehouse Childhood Cancer Initiative to determine CD8^+^ T-cell infiltration estimates across 20 available pediatric cancer types (Extended Data Fig. 4a). Choroid plexus carcinomas and medulloblastomas exhibited the lowest median CD8^+^ T-cell infiltration (0.002 and 0.005, respectively), while testicular germ cell tumors and lymphomas showed the highest (median 0.28 and 0.21, respectively). To determine whether the CD8 gene signature may also harbor prognostic value for pediatric patients with cancer agnostic of therapy, we performed survival analyses on three cancer types with available event-free survival (EFS) data through NCI TARGET. As previously published^9^, high levels of CD8^+^ T-cell estimates were prognostic of improved EFS in osteosarcoma (log-rank test, *P* = 0.04; Extended Data Fig. 4b), suggesting that there is an inflammatory subset of patients with osteosarcoma prone to longer survival. We did not, however, find any significant associations between EFS and CD8^+^ T-cell estimates in NBL or Wilms tumor (log-rank tests, *P* = 0.2 and 0.9, respectively; Extended Data Fig. 4c,d). Together, these results suggest that there is both an ICI-predictive, as well as prognostic value to the CD8 gene signature and highlight disease-specific associations in patients not treated with ICIs.

### High PD-L1 protein and gene expression were associated with improved PFS

**Fig. 3:**
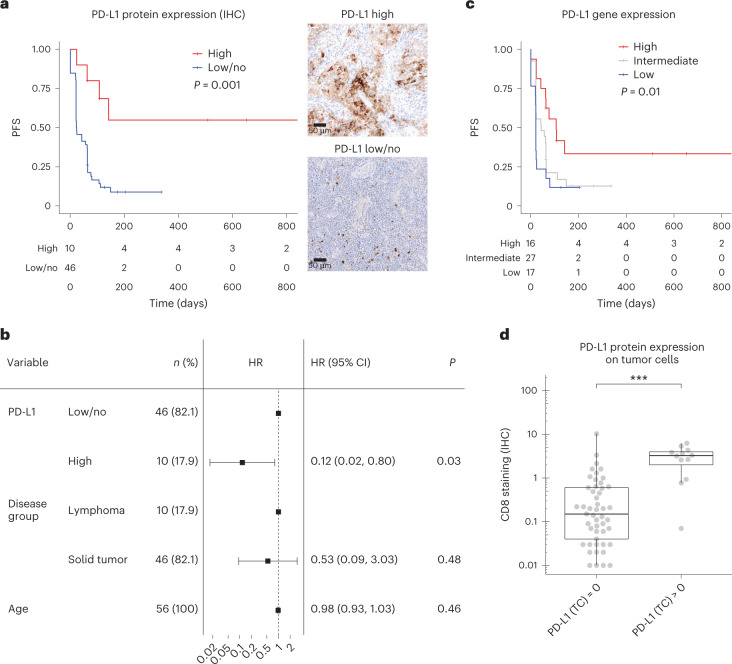
Baseline PD-L1 protein expression is associated with PFS in children treated with atezolizumab. **a**, Kaplan–Meier analysis of baseline tissue sample PD-L1 protein expression comparing PD-L1 high versus low/no expression groups (log-rank test, *n* = 56 patients). Exemplar images of a rhabdoid tumor with high expression of PD-L1 and a Wilms tumor with no expression on tumor cells are shown on the right. **b**, Cox proportional hazards model adjusting for disease group (lymphoma and solid tumors) and age, using PD-L1 protein expression as a predictor (two-sided Wald test, *n* = 56 patients). Center indicates HR and lines indicate CI. **c**, Kaplan–Meier analysis of *CD274* gene expression encoding PD-L1 dichotomized using quartiles (high, ≥75th percentile; intermediate, 25th–75th percentile; low, ≤25th percentile) (log-rank test, *n* = 60 patients). **d**, Box plot showing levels of CD8 staining (IHC) in 61 samples with PD-L1 expression on tumor cells compared to no expression (two-sided Student’s *t*-test, ***P < 0.001). Boxes show median and interquartile range (IQR) and whiskers represent 1.5 × IQR.

Next, we asked whether expression of PD-L1 as the primary drug target is associated with improved PFS in children. PD-L1 IHC was performed on available baseline tumor samples from iMATRIX-atezo. PD-L1 expression on immune cells ranged from 0 to 60% (median 0.5%) and was higher in lymphoma versus solid tumors (Fig. 1; medians 15% and 0.5%, respectively). Of 62 samples analyzed, 49 showed no expression of PD-L1 on tumor cells. Lymphomas displayed overall higher expression of PD-L1 on tumor cells than solid tumors (medians 50% and 0%, respectively). Patients whose samples harbored high PD-L1 expression exhibited improved PFS compared to low/no expression (log-rank test, *P* = 0.001; Fig. 3a). Cox proportional hazards model adjusted for cancer group (lymphoma versus solid tumors) indicated improved PFS for patients with samples categorized as PD-L1 high versus low/no expression (HR 0.12, 95% CI 0.02–0.8, *P* = 0.03; Fig. 3B). *CD274* expression encoding for PD-L1 protein was also positively associated with PFS (log-rank test, *P* = 0.01; Fig. 3c). Samples with PD-L1 protein expression on tumor cells had significantly higher levels of CD8 staining suggesting elevated expression of PD-L1 as a mode of immune evasion (Student’s *t*-test, *P* = 0.0007; Fig. 3d).

### High intratumoral TCRβ diversity was associated with improved PFS

**Fig. 4:**
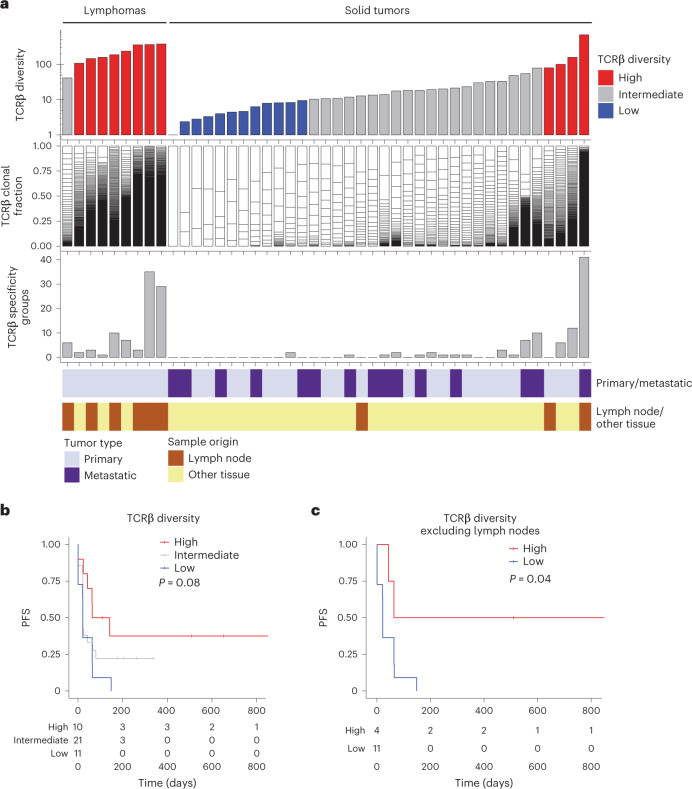
Baseline TCRβ diversity is associated with PFS in children treated with atezolizumab. **a**, Overview of TCRβ diversity, clonal distribution and TCR-specific motifs in lymphomas and solid tumors from the iMATRIX-atezo dataset (*n* = 45 samples). Red and blue denote high and low diversity groups (as in **b**). **b**, Kaplan–Meier analysis of TCRβ diversity in all tumor samples. TCRβ diversity is dichotomized using quartiles (high, ≥75th percentile; intermediate, 25th-75th percentile; low, ≤25th percentile; *n* = 42 patients). **c**, Kaplan–Meier analysis of high versus low TCRβ diversity (as in **b**) in tumor samples excluding those from lymph nodes (log-rank test, *n* = 15 patients).

**Extended Data Fig. 5:**
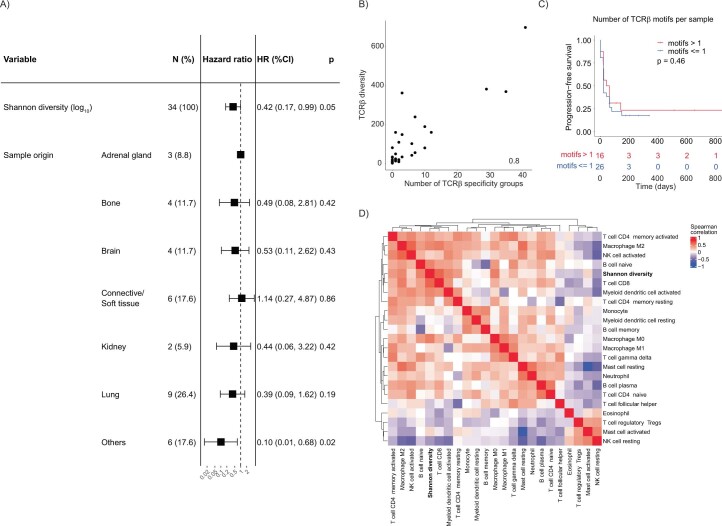
TCRβ diversity, but not number of TCR-specific motifs, shows a trend toward improved PFS in children treated with atezolizumab. (**A**) Multivariable analysis using Cox proportional hazards model adjusted for sample origin excluding those that originated from lymph nodes (two-sided Wald test, N = 34 patients). Center indicates hazard ratio and lines indicate confidence intervals. (**B**) TCRβ diversity is positively correlated with the number of TCRβ-specific motifs derived using the GLIPH2 algorithm. Value denotes Spearman r (N = 45 samples). (**C**) Kaplan–Meier analysis revealed no significant associations between PFS and the number of TCRβ-specific motifs (log-rank test, N = 42 patients). (**D**) Heat map depicting Spearman correlations among TCRβ diversity and immune-cell estimates from CIBERSORT in solid tumors excluding samples taken from lymph nodes (N = 33 samples). TCRβ diversity is shown in bold.

We next investigated whether immune diversity at baseline may be associated with PFS. From CapTCR-seq^5^ of 45 pre-treatment tumor tissue samples from patients enrolled into iMATRIX-atezo, we recovered 6,247 rearranged TCRβ sequences with a median length of 15 amino acids, of which 13 were considered public (occurring in more than one sample). TCRβ diversity as measured by the Shannon diversity index ranged from 1 to 697.2 and was higher in lymphomas than solid tumors (medians 188.5 and 13.7, respectively; Fig. 4a). We found no significant difference in TCRβ diversity between metastatic or primary tumor samples (medians 13.5 and 31.5, respectively, rank sum test, *P* = 0.08). As expected, samples that originated from lymph nodes had significantly higher TCRβ diversity than those from other tissues (medians 188.5 and 15.8, respectively, rank sum test, *P* = 0.0001). We identified four solid tumor samples with highly diverse T-cell repertoires: NBL, alveolar RMS, yolk sac tumor and one rare unspecified tumor. We clustered TCRβ sequences with similar antigen specificity^10^ and found 456 TCRβ motifs with an average of four motifs per sample (range 0–41) (Fig. 4a). From a univariate analysis, patients whose samples had a TCRβ diversity score at or above the upper quartile (≥80.3) showed a trend toward longer PFS compared to patients whose samples harbored interquartile and first quartile levels of TCRβ diversity (log-rank test, *P* = 0.08; Fig. 4b). The positive association with PFS between high and low diversity groups remained upon exclusion of lymph node samples (log-rank test, *P* = 0.04; Fig. 4c). Additionally, we excluded such samples and performed a multivariable analysis using Cox proportional hazards model adjusted for sample origin. We found a trend toward improved PFS in samples with higher levels of TCRβ diversity (HR 0.42, 95% CI 0.17–0.99, *P* = 0.05; Extended Data Fig. 5a). We found a high correlation between the number of TCRβ clusters and overall diversity (Spearman *r* = 0.8; Extended Data Fig. 5b), but no associations between the number of TCRβ motifs and PFS (Extended Data Fig. 5c). Correlating TCRβ diversity with immune cell-type estimates (CIBERSORT) in solid tumors revealed moderate to high correlations with CD8^+^ T cells, naive B cells and myeloid dendritic cells (Spearman *r* = 0.61, 0.47 and 0.46, respectively; Extended Data Fig. 5d) suggesting distinct co-varying immune cells associated with T-cell diversity. These data demonstrate that a diverse infiltrating T-cell repertoire in solid tumor tissues may serve as important exploratory biomarkers associated with prolonged treatment duration on atezolizumab.

### Panel-based TMB was not associated with PFS

**Extended Data Fig. 6:**
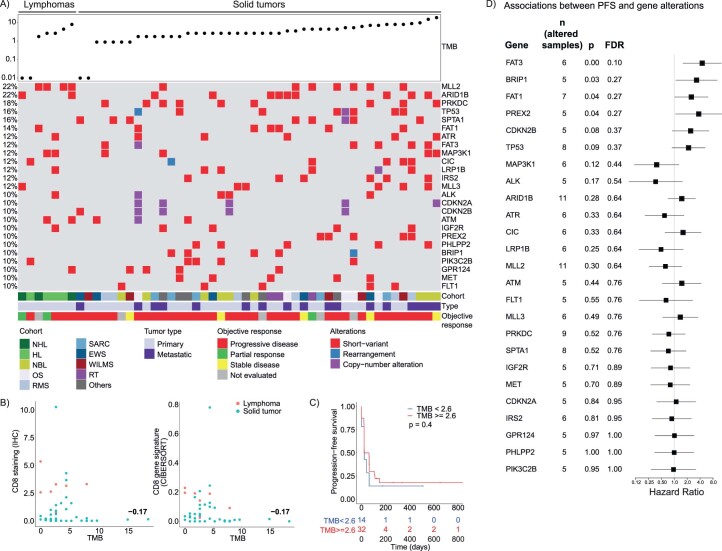
Neither panel-based TMB nor specific altered genes are prognostic of outcomes in pediatric patients treated with atezolizumab. (**A**) TMB and common genetic alterations occurring in >10% of samples in lymphomas and solid tumors as determined by the F1 assay (NHL: Non-Hodgkin lymphoma, HL: Hodgkin lymphoma; N = 51 samples). (**B**) Correlations between TMB (x-axes) and % CD8+ from IHC (y-axis, left panel) or CD8+ T-cell estimate from CIBERSORT (y-axis, right panel). Colors indicate lymphoma and solid tumor samples. Values denote Spearman r (N = 51 samples). (**C**) Kaplan–Meier analysis revealed TMB equal or more than median (≥2.6) was not significantly associated with improved PFS (log-rank test, N = 46 patients). (**D**) Associations of recurrently altered genes with PFS using univariable Cox proportional hazards model. Samples without mutations in the indicated genes were used as a reference group. HR < 1 indicates improved PFS in patients harboring gene alteration (two-sided Wald test, N = 46 patients). Center indicates hazard ratio and lines indicate confidence intervals.

We sought to determine the value of TMB as a predictive biomarker of response to ICI in pediatric cancers. TMB measured by the F1 CGP assay ranged from 0 to 18.4 mutations (mut) per Mb in 51 pre-treatment samples (median 2.6 mut per Mb; Extended Data Fig. 6a). In the iMATRIX-atezo dataset, only two patients, both with metastatic NBL, exhibited TMB > 10 mut per Mb; one patient experienced stable disease (SD), whereas the other had progressive disease (PD). We observed a statistical trend toward higher TMB in metastatic versus primary samples (medians 4.4 and 2.6 mut per Mb, respectively, *t*-test, *P* = 0.05). We did not find any significant correlation between TMB and the level of CD8^+^ T-cell infiltrate by IHC or estimated with CIBERSORT (Spearman *r* = −0.17; Extended Data Fig. 6b). Because the dynamic range of TMB in this dataset is insufficient to analyze outcomes between patients with tumors harboring TMB ≥ 10 versus <10 mut per Mb, we compared patients with tumors harboring TMB above or below the observed median (2.6 mut per Mb), but saw no difference in PFS (log-rank test, *P* = 0.4; Extended Data Fig. 6c).

While acknowledging the heterogeneity of cancer types in our cohort, we investigated whether recurrently altered genes were associated with PFS; we included short variants, insertion/deletions, rearrangements and copy number alterations. We found 25 genes that were altered in >10% of samples. Reflective of previous reports in pediatric and adult cancers, most recurrently altered genes included the chromatin remodeling gene, *ARID1B*^11,12^ (*n* = 12), a histone methyltransferase, *MLL2* (*KMT2D*)^13,14^ (*n* = 11) and the catalytic subunit of the DNA-dependent protein kinase, *PRKDC*^15^ (*n* = 9) (Extended Data Fig. 6a). We did not uncover any recurrently altered genes across cancer types that were associated with improved PFS (Extended Data Fig. 6d and Supplementary Table 2). While we cannot discount the possibility of mutations within specific cancer types mediating response, our results suggest tumor-intrinsic genetic aberrations are inferior determinants of immune response in basket trials such as iMATRIX-atezo.

### Differential gene expression and network analysis suggested biological determinants of immune response

**Fig. 5:**
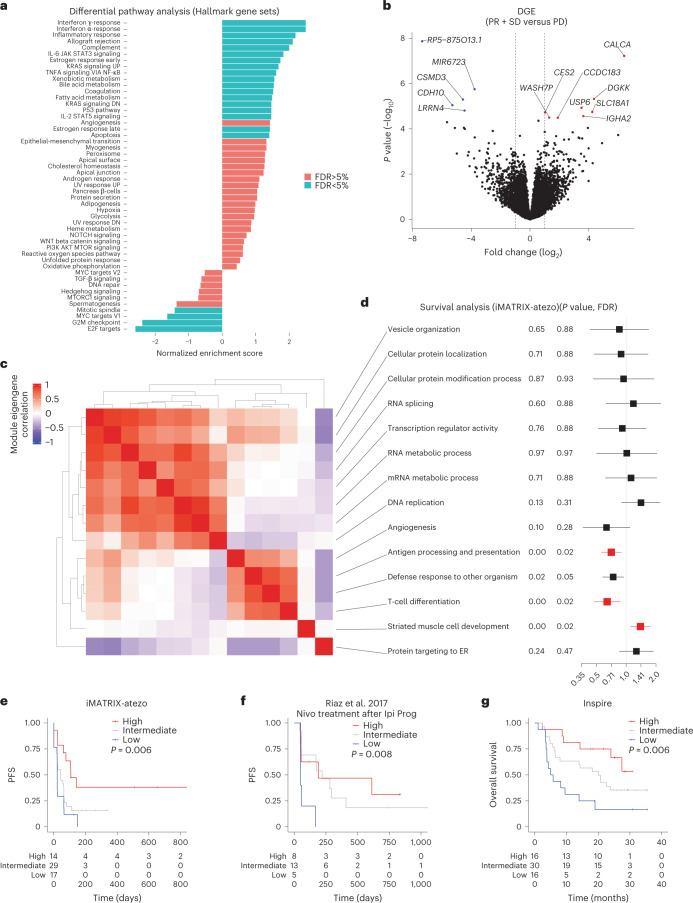
Differential gene expression and gene co-expression network reveal biological insights into atezolizumab clinical activity. **a**, Differential pathway enrichment scores for Hallmark gene set pathways using differentially expressed genes between patients who experienced PR/SD and PD (*n* = 60 patients). IL, interleukin; TGF, transforming growth factor. **b**, Volcano plot depicting top up- and downregulated genes in patients experiencing PR/SD compared to those with PD (*n* = 60 patients). Genes with absolute log_2_ fold change >1 (dashed lines) and FDR < 5% are denoted (two-sided Wald test). **c**, Heat map of consensus module eigengenes of 3,044 genes in samples spanning seven cancer types obtained from the Treehouse Childhood Cancer Initiative (*n* = 757 samples). **d**, We performed GO pathway analysis of the gene modules identified in **c** and determined the prognostic value of the corresponding pathways through the univariable Cox proportional hazards model utilizing the iMATRIX-atezo dataset (two-sided Wald test, *n* = 60 patients). Center indicates HR and lines indicate CI. Red bars denote gene modules with significant prognostic association at FDR < 5%. ER, endoplasmic reticulum. **e**–**g**, Kaplan–Meier analyses using enrichment scores obtained from ten core genes in the T-cell differentiation and antigen-presentation modules (PedCap score, from **c**,**d**) as predictors in the iMATRIX-atezo dataset (*n* = 60 patients, **e**), the CA209-038 trial of adult patients with melanoma treated with nivolumab following progression on ipilimumab (*n* = 26 patients, **f**) and the INSPIRE clinical trial of pembrolizumab in adult patients with solid tumors (*n* = 62 patients, **g**). Values are dichotomized using quartiles in each analysis (high, ≥75th percentile; intermediate, 25th-75th percentile; low, ≤25th percentile) (log-rank test). Source data

**Extended Data Fig. 7:**
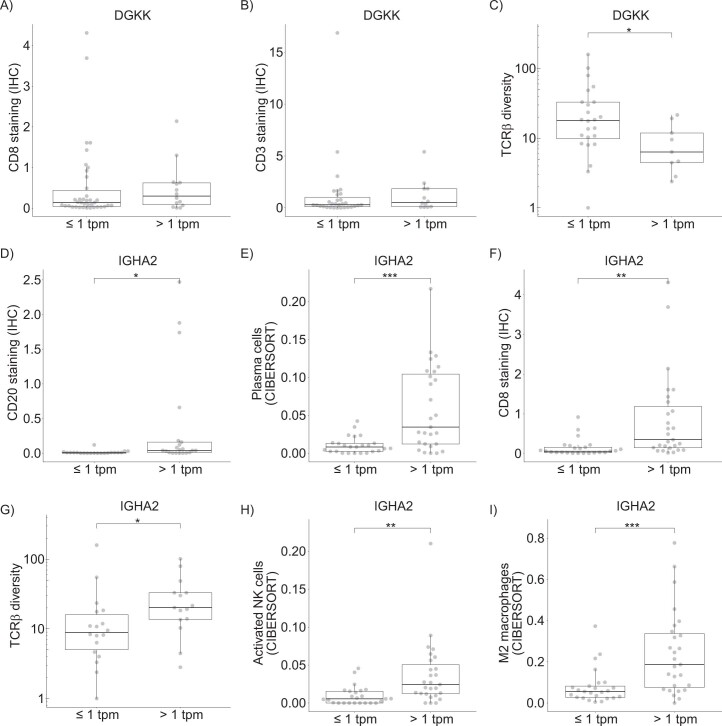
Top differentially up-regulated genes associated with distinct microenvironmental features in solid tumors. A-B) Levels of %CD8+ (N = 52 samples) (**A**) or %CD3+ (N = 43 samples) (**B**) determined using IHC in solid tumors expressing *DGKK* (>1 TPM) compared to non-expressing samples (≤1 TPM) (two-sided Student’s t-test, not significant). (**C**) Levels of log-transformed TCRβ diversity in solid tumors expressing *DGKK* (>1 TPM) compared to non-expressing samples (≤1 TPM, two-sided Student’s t-test, *p < 0.05) (N = 33 samples). (**D**, **E**) Levels of %CD20+ determined using IHC (N = 42 samples) (**D**) or plasma cell estimates obtained from CIBERSORT (N = 52 samples) (**E**) in solid tumor samples expressing *IGHA2* (>1 TPM) compared to non-expressing samples (≤1 TPM, two-sided Student’s t-test, *p < 0.05, ***p < 0.001). (**F**) Levels of %CD8+ in solid tumor samples expressing *IGHA2* (>1 TPM) compared to non-expressing samples (≤1 TPM, two-sided Student’s t-test, **p < 0.01) (N = 52 samples). (**G**) Levels of log-transformed TCRβ diversity in solid tumor samples expressing *IGHA2* (>1 TPM) compared to non-expressing samples (≤1 TPM, two-sided Student’s t-test, *p < 0.05) (N = 33 samples). (**H**–**I**) Levels of activated NK-cell (**H**) or M2 macrophage (**I**) estimates obtained from CIBERSORT in solid tumor samples expressing *IGHA2* (>1 TPM) compared to non-expressing samples (≤1 TPM, two-sided Student’s t-test, **p < 0.01, ***p < 0.001) (N = 52 samples). Boxes show median and interquartile range (IQR) and whiskers represent 1.5 × IQR. All indicated sample sizes represent biologically independent samples.

To identify molecular pathways associated with atezolizumab activity, we performed differential gene expression (DGE) analysis comparing patients experiencing partial response (PR)/SD and PD. DGE pathway analysis using the Hallmark gene sets revealed immune-related pathways upregulated in patients with PR/SD (Fig. 5a). The top highly expressed gene was calcitonin-related polypeptide alpha (*CALCA*), which encodes preprocalcitonin (Fig. 5b). According to the Human Protein Atlas database, preprocalcitonin is expressed in healthy bronchus and thyroid gland tissues, but is also expressed in lung cancers. For patients with melanoma, preprocalcitonin expression has been cited as a predictive biomarker of response to anti-PD-1 therapy^16^. A preprocalcitonin-derived peptide can act as a tumor neoantigen in cancers with altered antigen processing machinery and is recognized by specific T-cell clones^17,18^. The coiled-coil domain containing 183 (*CCDC183*) gene is primarily expressed in human testis, suggesting it may act as a cancer testis antigen. Diacylglycerol (DAG) kinase kappa (*DGKK*) was highly expressed in patients with PR/SD compared to PD (Fig. 5b). Although less is known about this gene, two DAG kinases (DGK-α and DGK-ζ) are known negative regulators of TCR signaling^19^. While there was no significant difference in CD8 or CD3 staining in solid tumors expressing *DGKK* (>1 transcripts per million (TPM)) versus those with no expression (≤1 TPM) (Student’s *t*-test, *P* = 0.87 and 0.92, respectively, Extended Data Fig. 7a,b), *DGKK*-expressing samples had significantly lower TCRβ diversity suggesting *DGKK* expression in antigen-exposed T cells (Student’s *t*-test, *P* = 0.02; Extended Data Fig. 7c). *IGHA2* encoding for immunoglobulin heavy chain α2 (IgA2) is primarily expressed by B-cell lineage cells, specifically, plasma cells. Solid tumors with *IGHA2* expression (>1 TPM) showed significantly higher levels of CD20 staining or plasma-cell estimates derived from CIBERSORT (Student’s *t*-test, *P* = 0.04 and 0.0001, respectively; Extended Data Fig. 7d,e). We found significantly higher levels of CD8 staining as well as TCRβ diversity in samples with *IGHA2* expression compared to those without (Student’s *t*-test, *P* = 0.003 and 0.04, respectively; Extended Data Fig. 7f,g). *IGHA2*-expressing samples also harbored significantly higher levels of activated natural killer (NK) cells or M2 macrophages as inferred using CIBERSORT (Student’s *t*-test, *P* = 0.003 and 0.0006, respectively; Extended Data Fig. 7h,i). These findings uncover potential tumor antigens and microenvironmental features in pediatric solid tumors that may impact response to ICIs.

We next sought to identify tumor-agnostic microenvironmental pathways associated with PFS using gene co-expression network analysis. We returned to the Treehouse dataset to perform a weighted co-expression network analysis^20^ using samples corresponding to the seven cancer types included in iMATRIX-atezo. We identified 3,044 genes that formed 14 gene modules that were common across the seven cancer types (Fig. 5c). Analysis of Gene Ontology (GO) pathways corresponding to the 14 gene modules revealed an enrichment of multiple pathways, including RNA processing, RNA metabolism, and immune response processes (Fig. 5c). When mapped back to the iMATRIX-atezo dataset, two gene modules, both involved in the immune system, were significantly associated with improved PFS (univariable Cox proportional hazards model, FDR < 5%; Fig. 5d and Supplementary Table 3). One module consisted of 28 genes related to antigen presentation and processing including HLAs, β-2-microglobulin (*B2M*), *TAP1* and members of the immunoproteasome (*PSMB8* and *PSMB9*). The second module comprised 58 co-expressed genes related to T-cell and NK-cell characteristics, including cytotoxicity (*GZMA*, *GZMH*, *GZMK* and *PRF1*), CD3 complex (*CD3E*, *CD3D*, *CD3G* and *CD247*), cell surface receptors (*CD8A* and *CD96*), and cytokines/chemokines (*CXCL9*, *CXCR3*, *CCL17*, *CCR4* and *IL7R*). We also identified a module of 28 genes that, when mapped back to the iMATRIX-atezo dataset, was inversely associated with PFS (Fig. 5d). This module consisted of genes involved in myogenesis, including myosin light chains (*MYL1* and *MYL2*), myogenin (*MYOG*) and members of the Klech-like family (*KLHL30* and *KLHL41*). The function and cell(s) of origin expressing these genes in the tumor microenvironment are not well characterized. One study found that myosin activity in melanoma cells adjacent to blood vessels induces protumorigenic macrophages via secreting chemokines and cytokines^21^. While our analysis may be confounded by heterogeneous sample origins and the use of bulk RNA-seq, the complement with the above recent finding hints that, in pediatric cancers, myogenesis may confer an immunosuppressive myeloid-like microenvironment.

**Extended Data Fig. 8:**
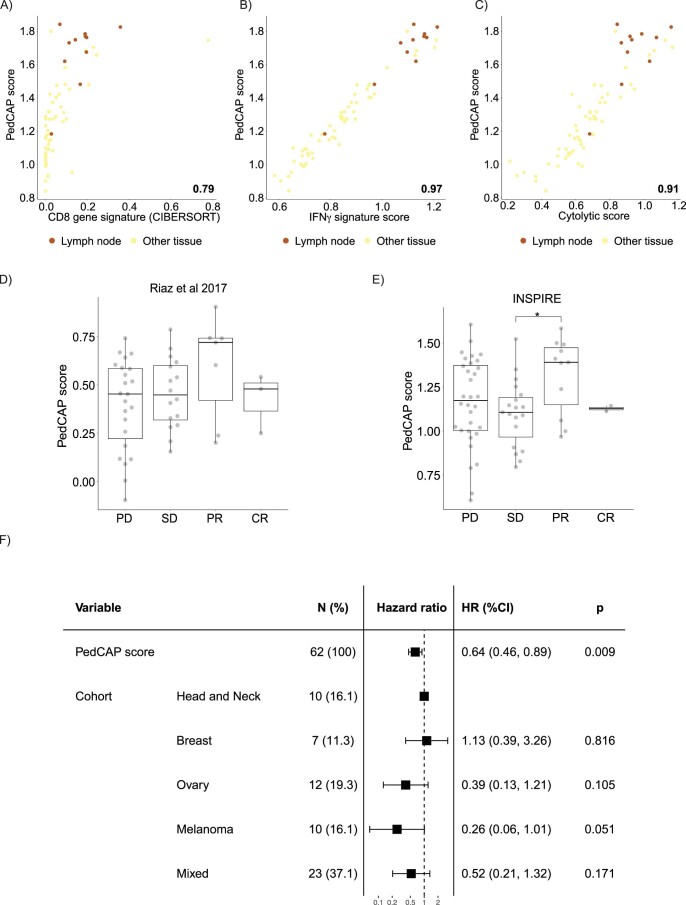
Enrichment scores of PedCAP is associated with objective response in two independent studies of adult cancer patients treated with ICI. (**A**–**C**) Correlation of PedCAP scores with CD8 gene signature derived from CIBERSORT (A, N = 66 samples), published interferon-γ signature (**B**, N = 66 samples) and cytolytic score (**C**, N = 66 samples). Values denote Spearman r. Colors denote sample origin as lymph node or one of the following: lung, soft tissues, bone, adrenal gland, kidney, brain, or unannotated tissue types. (**D**) PedCAP scores were significantly higher in patients experiencing partial response compared to patients experiencing progressive disease in the CA209-038 trial of nivolumab following progression on ipilimumab in adult patients with advanced melanoma (two-sided Student’s t-test, N = 49 samples). (**E**) PedCAP scores were significantly higher in patients experiencing partial response compared to those experiencing stable disease in the INSPIRE trial of pembrolizumab in adult patients with advanced solid tumors (two-sided Student’s t-test, *p < 0.05, N = 64 samples) (CR: complete response, PR; partial response, SD: stable disease, PD: progressive disease). (**F**) Multivariable Cox proportional hazards model adjusted for cohort indicates that higher PedCAP scores are associated with prolonged overall survival in the INSPIRE study (two-sided Wald test, N = 62 patients). Center indicates hazard ratio and lines indicate confidence intervals. In boxplots, boxes show median and interquartile range (IQR) and whiskers represent 1.5 × IQR. All indicated sample sizes represent biologically independent samples.

To identify the core genes responsible for driving these potentially predictive phenotypes within the two positively prognostic modules independent of cancer type, we determined correlations of gene expression in each cancer type with their corresponding module eigengene. This effort identified a ten-gene, highly correlated, tumor-agnostic gene set inclusive of three genes from the antigen-presentation module (*HLA-B*, *HLA-F* and *PSMB9*) and seven from the cytotoxic immune module (*CD2*, *CD3D*, *CD3E*, *CD3G*, *CXCR3*, *THEMIS* and *UBASH3A*), which we have termed the ‘Pediatric Cytotoxicity and Antigen Presentation’ (PedCAP) gene set (Supplementary Table 4). While the enrichment scores of the PedCAP gene set were highly correlated with T-cell frequencies as inferred by CIBERSORT (Spearman *r* = 0.79; Extended Data Fig. 8a), functional states reflective of an inflamed microenvironment seem to be even more tightly linked, as evidenced by high correlations with published interferon-γ signature^22^ and cytolytic scores^23^ (Spearman *r* = 0.97 and 0.91, respectively; Extended Data Fig. 8b,c). Higher levels of the PedCAP score in the iMATRIX-atezo dataset were associated with improved PFS (log-rank test, *P* = 0.006; Fig. 5e). To validate the prognostic value of the PedCAP scores in ICI therapy, we leveraged data from the trial of nivolumab in adults with melanoma who were ipilimumab-naive or had progressed on ipilimumab (CA209-038)^24^; samples from patients who experienced a PR on nivolumab had significantly higher PedCAP scores than those who had PD (Student’s *t*-test, *P* = 0.04; Extended Data Fig. 8d). Similarly, a high PedCAP score was associated with improved PFS in patients treated with nivolumab who had progressed on ipilimumab (log-rank test, *P* = 0.008; Fig. 5f). In the INSPIRE study of pembrolizumab in patients with advanced solid tumors^25^, samples from patients with PR had significantly higher scores than those with SD (Student’s *t*-test, *P* = 0.01; Extended Data Fig. 8e). Patients with a high PedCAP score exhibited improved overall survival compared to those with an intermediate/low score (log-rank test, *P* = 0.006; Fig. 5g). In a multivariable Cox proportional hazards model adjusting for cancer type, high PedCAP scores were associated with a lower HR (HR 0.6, 95% CI 0.46–0.89, *P* = 0.009; Extended Data Fig. 8f).

## Discussion

We report on the exploratory immunogenomic biomarker analysis of tumor samples from children with refractory/relapsed cancers before treatment with the anti-PD-L1 antibody, atezolizumab, in the iMATRIX-atezo study^4^. In iMATRIX-atezo, only 4 of 90 patients experienced a PR (4.4%); three with lymphoma and one with RT^4^. Similarly, in the ADVL1412 study of nivolumab ± ipilimumab, 4 of 85 patients (4.7%) achieved a PR (three with Hodgkin lymphoma and one with non-Hodgkin lymphoma)^2^. In the KEYNOTE-051 study of pembrolizumab in patients with PD-L1-positive tumors, 17 of 154 patients (11%) experienced a complete response/PR (nine with Hodgkin lymphoma and eight with a variety of pediatric solid tumors)^3^. These results demonstrate the potential for molecular biomarkers to improve outcomes and highlight the lack of robust tumor-agnostic biomarkers to identify patients with cancer who may benefit from ICI therapy.

One hurdle in the interpretation of PD-L1 as a robust biomarker is the spectrum of antibodies and scoring methodologies used, resulting in a lack of consensus in interpretation and applicability of PD-L1 to pediatric cancers^26,27^. In iMATRIX-atezo, high PD-L1 protein expression, determined using the SP142 antibody and defined as >5% in tumor cells or >10% in immune cells, was significantly associated with improved PFS. In KEYNOTE-051, PD-L1-positive tumors were identified using the 22C3 antibody and defined as >1% positivity in tumor cells or any positivity in stroma^3,28^; however, responses were still limited (17 of 154; 11%) indicating that PD-L1 is not sufficient as a standalone biomarker^3^. Although our data confirm PD-L1 as a relevant biomarker for pediatric cancer, cross-platform combinatorial biomarker analyses could potentially overcome drawbacks of the varied PD-L1 assays in future studies.

In iMATRIX-atezo, TMB > 2.6 mut per Mb (dataset median) measured by the F1 assay was not associated with higher levels of T cells or improved PFS. As pediatric cancers have generally lower overall mutation burden than adults^29^, our results are consistent with findings that TMB alone is not a sufficient predictive biomarker in adult renal cell carcinoma (median TMB = 1.1 mut per Mb)^30,31^. We hypothesize that TMB may only be clinically relevant in a subset of cancers with inherently high TMB such as adult melanoma and lung cancers^31^. Indeed, mutation burden was predictive of ICI response and associated with higher CD8^+^ T-cell infiltration in pediatric patients with ultra-hypermutant cancers (>100 mut per Mb)^32^. These cancers typically have somatic mutations in DNA polymerase genes *POLE* or *POLD1* that arise in patients with constitutional mismatch repair deficiency syndrome, which occurs due to germline mutations in DNA mismatch repair genes^33^. These data indicate that TMB is not a predictor of PFS in pediatric patients with non-hypermutant cancers treated with atezolizumab.

We explored infiltrating TCRβ diversity as a predictor of survival in pediatric patients treated with atezolizumab and observed a trend toward improved PFS with higher TCRβ diversity, but not with TCRβ specificity groups. Considering a report showing how different T-cell clonotypes targeting the same mutations evolve over time^34^, our results suggest that a polyclonal infiltrating T-cell repertoire may increase the probability of T-cell reactivity in response to atezolizumab. Analysis of baseline T-cell diversity in adult melanoma studies, however, show conflicting prognostic values. While two studies found that high T-cell clonality was associated with response to ICI^35,36^, Riaz et al. observed no significant difference in tumor-infiltrating lymphocyte diversity or clonality before treatment with nivolumab between responders and non-responders^24^. In the neoadjuvant setting, Amaria et al. reported that high T-cell diversity and clonality at baseline correlated with response to nivolumab, but not ipilimumab^37^. Although further validation in a larger cohort is warranted, our findings suggest that a diverse infiltrating T-cell repertoire may be predictive of response in pediatric patients treated with atezolizumab.

DGE analysis between patients with PR/SD versus PD provided biological insights into atezolizumab activity. The top highly upregulated gene, *CALCA*, encodes preprocalcitonin that generates a neoantigen in immune-evaded tumors^17,18^. A distinct class of tumor antigens called T-cell epitopes associated with impaired peptide processing (TEIPP) has been identified in tumors with transporter associated with antigen processing (TAP) deficiencies and low expression of MHC class I^38–40^. Enhanced levels of MHC class I can activate TEIPP-specific T cells in mice^41^. We speculate that atezolizumab may enhance T-cell response in *CALCA*-expressing cells with intact antigen-presentation machinery. Elevated expression of the testis-specific *CCDC183* gene in patients who experienced PR/SD suggests that a possible cancer testis antigen (CTA) may be present in pediatric cancers. CTAs are a source of neoantigens in a variety of cancers, including pediatric solid tumors. One study identified peptides derived from a CTA called PRAME (preferentially expressed antigen in melanoma) in OS and EWS and showed that PRAME-specific T cells can induce tumor regression^9^. Together, with the lack of associations between TMB and PFS in this dataset, our findings suggest that TEIPP and CTA may be tumor-intrinsic factors determining the level of antitumor immune activity in pediatric cancers.

Higher expression of *IGHA2* and its associations with higher levels of T cells, NK cells and M2 macrophages in solid tumors hinted at the presence of tertiary lymphoid structures (TLSs) in patients experiencing PR/SD compared to PD. B-cell signatures have been associated with ICI response in adults with melanoma and sarcoma^42–44^. In these studies, spatial profiling uncovered T- and B-cell interactions and distinct cellular phenotypes in the TLSs of these tumors^42–44^. One renal cell cancer study demonstrated that B-cell differentiation to plasma cells in TLS and immunoglobulins bound to apoptotic tumor cells were associated with ICI response^45^. Although spatial resolution analyses are lacking in pediatric cancers, our study suggests that the presence of TLS in pediatric solid tumors may be associated with antitumor immune responses.

The gene co-expression network analysis identified core pathways in the pediatric tumor microenvironment associated with improved PFS. PedCAP scores were associated with PFS in pediatric and adult cancers treated with ICI and consisted of genes critical for T-cell functions expressed in several cell types. These include genes encoding CD3 chains, CD2 co-stimulatory molecule and *UBASH3A*, a negative regulator of TCR signaling expressed in CD4^+^ and CD8^+^ T cells. Its paralog, *UBASH3B* is expressed in tumor-infiltrating CD8^+^ NK-like cells as well as CD8^+^ exhausted T cells^46^. We also found a chemokine receptor, *CXCR3* that is expressed in a variety of immune cell types. Its ligand *CXCL9* was predictive of ICI response in adult cancers in a meta-analysis^47^, suggesting that the CXCR3-CXCL9 axis plays a role in cancer immunity and mechanisms of ICI response^48^. Among antigen-presentation hub genes, we identified *HLA-F*, a non-classical MHC class I gene. HLA-F interacts with inhibitory and activating receptors on NK cells and in its tetramer form, is able to present peptides^49–51^. Together, this analysis identifies tumor-agnostic core molecular pathways with biological relevance associated with immune response in pediatric cancers.

Our work identified a series of potential biomarkers that can predict response to atezolizumab across pediatric cancers. Although they require validation in a larger dataset, our findings highlight the value of clinical genomics and RNA-seq to nominate patients for ICI therapy^52^. To integrate these biomarkers and following the cancer immunogram framework^53^, we propose a multimodal approach to evaluate a patient’s likelihood of benefiting from ICI therapy in prospective trials. We speculate that incorporating the cross-platform predictors we identified, including CD8 and PD-L1 protein expression, immunoglobulin and CTA gene expression and TCRβ diversity into a comprehensive model will aid clinicians in identifying candidates for ICI therapy.

## Methods

### iMATRIX-atezolizumab participants and samples

The iMATRIX-atezo clinical trial from which collected patient samples were analyzed for the exploratory study herein is registered with ClinicalTrials.gov at NCT02541604. The trial protocol can be accessed as part of the supplement to the primary clinical manuscript^4^. The iMATRIX-atezo study was conducted in accordance with the principles of the Declaration of Helsinki and Good Clinical Practice and was approved by the relevant ethics bodies at each participating site: in Denmark, Rigshospitalet Section of Paediatric Hematology and Oncology Adolescent Medicine by the central De Videnskabsetiske Komitéer Region Hovedstaden; in France, Institut de Cancerologie Gustave Roussy Département de Pediatrie, Institut Curie Département de Pediatrie and Centre Léon Bérard by the central Comité de Protection des Personnes Ile de France VII; in Germany, Universitätsklinikum Frankfurt Zentrum für Kinder und Jugendmedizin by the local Ethikkommission des Fachbereichs Medizin der Johann Wolfgang Goethe-Universitat Frankfurt; in Israel, Schneider Children’s Medical Center of Israel Department of Pediatric Hematology and Oncology by the local Ethic (Helsinki) Committee at Rabin Medical Center; in Italy, Ospedale Pediatrico Bambino Gesù Dipartimento di Onco-Ematologia Pediatrica e Medicina Trasfusionale by the local Fondazione Instituto de Ricovero e Cura a Carattere Scientifico (IRCCS) Ospedale Pediatrico Bambino Gesù, Azienda Ospedaliero-Universitaria di Padova Onco-Ematologia Pediatrica by the local Comitato Etico per Sperimentazione Clinica della Provincia di Padova, lstituto Pediatrico di Ricovero e Cura a Carattere Scientifico lstituto Giannina Gaslini by the local Comitato Etico Regione Liguria Sezione 3 in Genova, Azienda Ospedaliera Universitaria Citta della Salute e della Scienza di Torino by the local Comitato Etico Interaziendale in Torino and Instituto Nazionale dei Tumori by the local Comitato Etico della Fondazione IRCCS in Milan; in the Netherlands, Erasmus University Medical Center (MC) by the central Erasmus MC Medical Ethics Review Committee; in Spain, Hospital lnfantil Universitario Nino Jesus by the central al Comité de Ética de Investigación Clínicas (CEIC) Nino Jesus, Hospital Universitario Vall d’Hebron by the local Unidad de Soporte al CEIC and Hospital San Joan de Deu Unitat de Recerca Clinica by the local CEIC; in Switzerland, University Children’s Hospital Zurich by the central Kantonale Ethikkommission Zurich; in the United Kingdom, Leeds General Infirmary Leeds Children’s Hospital Great George, The Royal Marsden National Health Service (NHS) Foundation Trust, Birmingham Women’s and Children’s NGS Foundation Trust Birmingham Children’s Hospital, Bristol Royal Hospital for Children by the central NRES Committee South-Central Berkshire; and in the United States, each with their corresponding local ethics committee, Arkansas Children’s Hospital, Penn State Hershey Children’s Hospital, Dana Farber Cancer Institute, MD Anderson Cancer Center Division of Pediatrics, Stanford University School of Medicine Department of Pediatric Hematology and Oncology, University of Texas Health Sciences Center at San Antonio and Memorial Sloan Kettering Cancer Center. An independent data monitoring committee monitored patient safety throughout the study.

As previously described^4^, the iMATRIX-atezo clinical trial was designed by the sponsor (F. Hoffmann-La Roche) and the clinical trial investigators. The trial sponsors F. Hoffmann-La Roche and Genentech were involved in the administration and conduct of study procedures, coordination of data collection, data analysis and data interpretation. During the trial enrollment period (November 2015 to April 2018), individual patient data were collected by the clinical trial investigators and their research teams at their respective institutions and were interpreted by the sponsor and investigators. The primary endpoints of the iMATRIX-atezo clinical trial were the safety (assessed by incidences of adverse events) and pharmacokinetics (assessed by serum concentrations) of atezolizumab. Secondary end points were preliminary antitumor activity of atezolizumab (proportion of patients achieving an objective response, clinical benefit and PFS, including duration of objective response and overall survival) and immunogenicity of atezolizumab (frequency of treatment-emergent anti-drug antibodies relative to anti-drug antibody prevalence at baseline). Correlation between response to atezolizumab and PD-L1 tumor expression was a prespecified exploratory end point.

From 28 centers across ten countries, a total of 90 patients were enrolled in the phase 1–2 iMATRIX-atezo clinical trial. Patients were enrolled regardless of sex (sex was self-reported via electronic data capture by clinical trial site staff). Written informed consent was obtained before study participation from each participant, including consent for the collection and use of patients’ samples. Consent was not provided to share sex data in a disaggregated (patient-level) manner and no analyses by sex were conducted as part of the planned clinical study analyses or the study herein. Patients in the safety-evaluable population (*n* = 87) received a median of two doses (range 1–39), with a median duration of exposure to atezolizumab of 0.8 months (range 0–29.2) and a median total cumulative dose of 1,980 mg (range 132–37,170). A total of 84 (96.6%) events occurred during the study. Disease progression was reported in 73 patients and 11 patients died. The median duration of PFS across all cohorts was 1.3 months (95% CI 1.2–1.4). Censor was defined as no progression of disease or death up to the clinical cutoff date for the primary clinical analysis.

Patient samples and sample data (IHC, FoundationOne (F1) sequencing and whole transcriptome sequencing) were shared as appropriate and in accordance with these consents through a research collaboration agreement between Roche/Genentech and the Princess Margaret Cancer Centre. Overall, 75 tumor tissue samples were collected before treatment and were available for analysis. These samples were primarily archival samples or were collected through resection or biopsy during the patient’s screening period for the trial. These samples are termed as ‘pre-treatment’. Eight samples were removed due to failed library preparation or sequencing quality control. We included 66 unique tumor samples in analyses and results described herein (Supplementary Table 1). Disease groups were defined as previously reported^4^ with minor modifications. Hodgkin and non-Hodgkin lymphomas were grouped into lymphoma. Non-RMS soft tissue sarcoma consisting of a variety of sarcomas was grouped in mixed SARC and included Ewing-like CIC-DUX4 fusion^+^ sarcoma, desmoplastic small round cell tumor, synovial sarcoma, clear cell sarcoma and malignant peripheral nerve sheath tumor. Rare tumor types not represented in the other enrollment cohorts, with or without previous literature documentation of PD-L1 positivity^3^, were grouped as ‘other’. Three cases of ATRT and one case of malignant RT that was initially enrolled in the non-RMS cohort^4^ were grouped into ATRT/RT.

### Immunohistochemistry

IHC was performed retrospectively in batch during the course of the iMATRIX-atezo clinical trial on patient tumor tissue samples at Ventana for PD-L1 (*n* = 61) and at HistoGeneX (now known as CellCarta) for CD3 (*n* = 57), CD8 (*n* = 66) and CD20 (*n* = 54). Owing to limited biospecimen availability, IHC staining was performed only once for each tumor sample. PD-L1 expression was categorized by tumor cell (TC) and tumor-infiltrating immune cell (IC) IHC scoring with the validated SP142 antibody assay (Ventana) as previously described^4^. Briefly, tumors were categorized as having high expression (TC 2–3, ≥5% PD-L1-positive tumor cells; IC 2–3, ≥10% PD-L1-positive immune infiltrates), low expression (TC1 or IC1, ≥1% to <5% PD-L1-positive tumor cells or immune infiltrates) or no expression (TC0 or IC0, <1% PD-L1-positive tumor cells or immune infiltrates). PD-L1 expression on tumor samples from patients with non-Hodgkin lymphoma or Hodgkin lymphoma were analyzed in the same manner as tumor samples from patients with solid tumors. The PD-L1 SP142 antibody assay was supplied at the manufacturer’s recommended concentration in a ready-to-use dispenser.

CD3 (clone 2GV6, Ventana cat. no. 790-4341, supplied at the proper concentration in a ready-to-use dispenser), CD8 (clone C8/144B, DAKO cat. no. M7103, diluted 1:75 to 2.1 µg ml^−1^ concentration) and CD20 (clone L26, Ventana cat. no. 760-2531, supplied at the proper concentration in a ready-to-use dispenser) were measured as a percentage of antibody-stained cells in the tumor center and in the invasive margin of the tumor. Specifically, CD3, CD8 or CD20 stained images were analyzed digitally with the image analysis software Definiens Architect or Definiens Developer (v.2.1.1) and Definiens Tissue Studio (v.3.6.1) to determine the amount of IHC staining by measuring the area of the IHC-stained cells or structure in the center of a tumor and in the invasive margin of the tumor. A central tumor region was delineated by a trained pathologist. At the interface between malignant and adjacent healthy tissue, a 1,000-µm wide border (invasive margin region) centered around the perimeter was drawn. To reduce analysis time, tiling followed by systematic random sampling of a minimum of 35% of the tiles, but no fewer than 100 tiles, was performed. For every region of interest (ROI), the relative area of marker-positive cells was calculated. This is the absolute area of the marker-positive cells in the sampled area detected by the algorithm and divided by the total number of ROIs in the sampled area.

### Comprehensive genomic profiling (FoundationOne or F1)

Genomic alterations and TMB in 52 pre-treatment tumor tissue samples were detected using the F1 targeted next-generation sequencing panel at a CLIA-certified, CAP-accredited reference laboratory (Foundation Medicine), as previously described^54,55^. The F1 panel version used herein sequences the entire coding regions of 395 cancer-related genes and select introns of 31 genes that are rearranged or altered in cancer^54^. TMB was defined as the number of somatic, coding, base substitution and indel mutations per Mb of genome examined and calculated as described previously^55,56^. We removed one sample with TMB score annotated as ‘not detected’.

### Whole transcriptome sequencing data processing

Whole transcriptome sequencing (RNA-seq) was performed on bulk RNA extracted from 75 baseline tumor tissue samples at Q Squared Solutions Expression Analysis. Sequencing libraries were generated using the Illumina TruSeq RNA Access method, a hybridization-based protocol to enrich for coding RNAs from total RNA-seq libraries. The method consists of two major steps: total RNA library preparation and coding RNA library enrichment. First-strand complementary DNA synthesis was primed from total RNA using random primers, followed by the generation of second-strand cDNA with dUTP (in place of dTTP) in a master mix. This facilitates the preservation of strand information, as amplification in subsequent steps will stall when it encounters uracil in the nucleotide strand. Double-stranded cDNA then underwent end repair, A-tailing and ligation of adaptors that include index sequences. One sample failed library preparation and was removed from the dataset. The resulting molecules were amplified via PCR, their yield and size distribution were determined using a BioAnalyzer and their concentrations were normalized in preparation for the enrichment step. Libraries were enriched for the messenger RNA fraction by positive selection using a cocktail of biotinylated oligonucleotides corresponding to coding regions of the genome. Targeted library molecules were then captured through the hybridized biotinylated oligonucleotide probe using streptavidin-conjugated beads. After two rounds of hybridization/capture reactions, the enriched library molecules were PCR amplified, quantified, then normalized and pooled in preparation for sequencing on an Illumina HiSeq 2500. Libraries were sequenced using the Illumina sequencing-by-synthesis platform on an Illumina HiSeq 2500 at a paired-end read length of 50 bp and a total read depth of 50 million reads per sample. Seven samples failed sequencing quality control and were removed from further analysis.

RNA-seq raw reads were aligned to the human genome reference (hg38) using STAR aligner v.2.4.2a with default settings^57^ and gene expression was quantified using RSEM v.1.2.29 (ref. ^58^) with GENCODE v.21. We used log_2_ transformed TPM values for gene expression and co-expression network analyses.

### Immune deconvolution analysis

Comprehensive immune deconvolution analysis was completed through the TIMER2 (ref. ^6^) web portal (http://timer.cistrome.org/) using immunedeconv v.2.0.0 (ref. ^59^) R package, which integrates six published algorithms: CIBERSORT v1.0.4 (ref. ^7^), EPIC v.1.1.5 (ref. ^60^), MCPCounter v.1.2.0 (ref. ^8^), quanTiseq v.3.16 (ref. ^61^), TIMER v.0.1 (ref. ^62^) and xCell v.1.10 (ref. ^63^). CD8^+^ T-cell estimates derived from each tool were correlated with the percentage of CD8^+^ cells obtained from IHC on the iMATRIX-atezo dataset.

### TCR sequencing

To study T-cell repertoire in tumor samples, we used hybrid capture TCR sequencing (CapTCR-seq^5^) to enrich for rearranged variable diversity joining regions. Briefly, DNA was extracted from tumor tissue samples using QIAGEN Allprep kit and sheared to reach median 250-bp insert size. Illumina DNA libraries were prepared from 120–500 ng DNA using the KAPA HyperPrep library preparation kit with custom adaptors, including 2-bp molecular identifiers^64^. We pooled two indexed libraries and performed hybrid capture using a custom TCR probeset^5^, following the Integrated DNA Technologies hybridization capture protocol. Captured DNA pools were then deep sequenced to a minimum of 1.6 million reads on the Illumina NovaSeq platform at the Princess Margaret Genomics Centre. We used the MiXCR v.2.1.12 immune repertoire pipeline^65^ to recover clonotype counts and sequences and analyzed immune diversity using the iNEXT v.2.0.20 R package^66^. Of 51 tumor samples, 5 were collected from time points other than baseline (pre-treatment) and one sample failed TCR-sequencing quality control. We therefore removed these samples from TCR analysis (*n* = 45).

### Differential gene expression analysis

For the purpose of DGE analysis, we compared gene expression profiles of patients experiencing PR or SD (*n* = 11) to PD (*n* = 49). We removed six samples with no clinical response information from this analysis. We used the tximport v.1.18.0 R package^67^ to import RSEM files and used the DESeq2 v.1.30.0 R package^68^ for DGE analysis. Given our heterogenous dataset, we controlled for sequencing batch, sample origin and disease type as covariates in the design. We used the fgsea v.1.16.0 R package^69^ for DGE pathway analysis.

### Co-expression network analysis

To identify co-expressed gene modules in a large number of pediatric cancer samples independent of cancer type, we performed consensus-weighted co-expression gene network analysis (WGCNA)^20^ using data from the Treehouse Childhood Cancer Initiative. We downloaded the log_2_-transformed gene expression matrix from the Treehouse portal. We focused our analysis on 835 samples from cancer types included in the iMATRIX-atezo study: lymphoma (*n* = 25), NBL (*n* = 201), OS (*n* = 84), RMS (*n* = 168), SARC (*n* = 40), Wilms tumor (*n* = 136) and EWS (*n* = 81), excluding rhabdoid tumors and ATRTs due to insufficient number of available samples. To reflect the sarcoma group in the iMATRIX-atezo study (SARC), we grouped mixed and unspecified sarcomas into the SARC group in this analysis: synovial sarcoma (*n* = 13), undifferentiated sarcoma not otherwise specified (*n* = 12), undifferentiated pleomorphic sarcoma (*n* = 4), epithelioid sarcoma (*n* = 3), undifferentiated spindle cell sarcoma (*n* = 2), INI-deficient soft tissue sarcoma not otherwise specified (*n* = 2), undifferentiated hepatic sarcoma (*n* = 2), alveolar soft part sarcoma (*n* = 1) and endometrial stromal sarcoma (*n* = 1). We subset genes to the 24,435 genes included in the iMATRIX-atezo dataset. Genes with no observed expression were removed in more than half of samples. A total of 19,010 genes were used as input. To remove outlier samples, we clustered samples in each cancer type and removed those with >75th percentile distance leading to the final 757 samples. For each cancer type, we first determined the appropriate soft threshold using the pickSoftThreshold function in the WGCNA v.1.69 R package. We chose the minimum threshold for each cancer type with the scale-free topology model fit, *R*^2^ > 0.8, as previously described^20^. We then calculated Pearson correlations for each cancer type and obtained signed adjacency matrices with the corresponding soft power. We obtained topological overlap matrices (TOMs) from adjacency matrices using the TOMsimilarity function in the WGCNA R package (type ‘unsigned’). We scaled TOMs and determined a consensus TOM by taking a parallel minimum of all seven cancer datasets. Gene modules were identified using the dynamic tree cut algorithm^70^ with a minimum cluster size of 20 and merged similar modules using the mergeCloseModules function in the WGCNA R package. This analysis identified 14 consensus modules across seven cancer types, including 3,044 genes. To identify clinical relevance of modules in the iMATRIX-atezo study, we used the average expression of genes within modules and performed univariable and multivariable survival analyses. To identify core genes in prognostic modules, we determined Pearson correlations of each gene with their module eigengene. We reported ten genes with an eigengene correlation of >0.8 as the most relevant pan-tumor genes and used un-normalized, single-sample gene set enrichment analysis in the GSVA v.1.38.2 R package to score samples.

### Statistics and reproducibility

No statistical method was used to predetermine sample size. The experiments were not randomized. The investigators were not blinded to allocation during experiments and outcome assessment. Eight samples were removed due to failed library preparation or sequencing quality control. For TMB analysis, one sample was removed with the TMB score annotated as ‘not detected’. For TCR-sequencing analysis, one sample was removed due to bad quality. Owing to limited biospecimen availability, IHC staining was performed only once for each tumor sample. All analyses were performed and plotted on R.4.0 in Jupyter notebooks. For survival analyses, as patients received atezolizumab until loss of clinical activity or disease progression^4^, we used duration of treatment as PFS. For survival analysis using the NCI TARGET data, we used ‘Event-Free Survival Time in Days’ and ‘First Event’ as reported in the metadata, with ‘Event’, ‘Death’, ‘Progression’, ‘Relapse’ and ‘Second Malignant Neoplasm’ recoded as 1. We used log-rank tests for Kaplan–Meier analyses using the survival v.3.2-7, survminer v.0.4.9 R packages. Cox proportional hazards models were fitted to adjust for covariates as described in the text using function coxph from the survival package v.3.2-7 in R. Data distributions for protein and gene expression comparisons were assumed to be normal but this was not formally tested. Plot esthetics were edited using Adobe Illustrator v.24.0.01.

### Reporting summary

Further information on research design is available in the Nature Portfolio Reporting Summary linked to this article.

## Supplementary information

Reporting Summary

Supplementary Table 1 Summary of the analysis dataset, including aggregate patient characteristics, sample types and testing performed.

Supplementary Table 2 Univariable Cox proportional hazards models for recurrently altered genes.

Supplementary Table 3 Univariable Cox proportional hazards models for co-expressed gene modules.

Supplementary Table 4 Correlation across genes and module eigengenes.

## Data Availability

Individual patient-level data, including de-identified clinical metadata, raw RNA-seq data, raw TCR-seq data and processed F1-sequencing data are made available to qualified researchers at the European Genome-Phenome Archive under accession no. EGAS00001006004. To request access to such data, researchers can contact devsci-dac-d@gene.com. The data will be released to such requesters with necessary agreements to enforce terms such as security, patient privacy and consent of specified data use, consistent with evolving, applicable data protection laws. For up-to-date details on Roche’s Global Policy on the Sharing of Clinical Information and how to request access to related clinical study documents, see https://go.roche.com/data_sharing. Tumor Compendium v.11 was downloaded from the Treehouse Childhood Cancer Initiative (https://treehousegenomics.soe.ucsc.edu/public-data/#tumor_v11_polyA). One-hundred and nine publicly available Sequence Read Archive files from the CA209-038 study^24^ (NCT01621490) were downloaded using the NCBI SRA selector (SRP094781). Source data for Fig. 5a–c and Extended Data Fig. 4a have been provided as Source Data files. All other data supporting the findings of this study are available from the corresponding author(s) on reasonable request. Clinical data for the CA209-038 study are available on GitHub (https://github.com/riazn/bms038_analysis/). Gene expression and clinical outcomes data from the INSPIRE trial are available as described before^25,71^. The reference human genome (hg38) was downloaded from https://genome.ucsc.edu/. Source data are provided with this paper.

## Source data

Source Data Fig. 5 First tab shows statistical source data for Fig. 5a,b. Second tab shows statistical source data for Fig. 5c.
Source Data Extended Data Fig. 4 Third tab shows statistical source data for Extended Data Fig. 4.

## Code availability

Custom codes to reproduce the analyses and figures are publicly available at https://github.com/pughlab/iMATRIX-Atezo_Biomarker.

## References

[1] Sharma P, Allison JP (2015). The future of immune checkpoint therapy. Science.

[2] Davis KL (2020). Nivolumab in children and young adults with relapsed or refractory solid tumours or lymphoma (ADVL1412): a multicentre, open-label, single-arm, phase 1–2 trial. Lancet Oncol..

[3] Geoerger B (2020). Pembrolizumab in paediatric patients with advanced melanoma or a PD-L1-positive, advanced, relapsed, or refractory solid tumour or lymphoma (KEYNOTE-051): interim analysis of an open-label, single-arm, phase 1–2 trial. Lancet Oncol..

[4] Geoerger B (2020). Atezolizumab for children and young adults with previously treated solid tumours, non-Hodgkin lymphoma, and Hodgkin lymphoma (iMATRIX): a multicentre phase 1–2 study. Lancet Oncol..

[5] Mulder DT (2018). CapTCR-seq: hybrid capture for T-cell receptor repertoire profiling. Blood Adv..

[6] Li T (2020). TIMER2.0 for analysis of tumor-infiltrating immune cells. Nucleic Acids Res..

[7] Newman AM (2015). Robust enumeration of cell subsets from tissue expression profiles. Nat. Methods.

[8] Becht E (2016). Estimating the population abundance of tissue-infiltrating immune and stromal cell populations using gene expression. Genome Biol..

[9] Brohl AS (2021). Immuno-transcriptomic profiling of extracranial pediatric solid malignancies. Cell Rep..

[10] Huang H, Wang C, Rubelt F, Scriba TJ, Davis MM (2020). Analyzing the *Mycobacterium tuberculosis* immune response by T-cell receptor clustering with GLIPH2 and genome-wide antigen screening. Nat. Biotechnol..

[11] Pugh TJ (2013). The genetic landscape of high-risk neuroblastoma. Nat. Genet..

[12] Sausen M (2013). Integrated genomic analyses identify ARID1A and ARID1B alterations in the childhood cancer neuroblastoma. Nat. Genet..

[13] Pugh TJ (2012). Medulloblastoma exome sequencing uncovers subtype-specific somatic mutations. Nature.

[14] Crompton BD (2014). The genomic landscape of pediatric Ewing sarcoma. Cancer Discov.

[15] Wang X (2008). Mutational analysis of thirty-two double-strand DNA break repair genes in breast and pancreatic cancers. Cancer Res..

[16] Gide TN (2019). Distinct immune cell populations define response to anti-PD-1 monotherapy and anti-PD-1/Anti-CTLA-4 combined therapy. Cancer Cell.

[17] El Hage F (2008). Preprocalcitonin signal peptide generates a cytotoxic T lymphocyte-defined tumor epitope processed by a proteasome-independent pathway. Proc. Natl Acad. Sci. USA.

[18] Durgeau A (2018). Human preprocalcitonin self-antigen generates TAP-dependent and -independent epitopes triggering optimised T-cell responses toward immune-escaped tumours. Nat. Commun..

[19] Olenchock BA (2006). Disruption of diacylglycerol metabolism impairs the induction of T cell anergy. Nat. Immunol..

[20] Zhang, B. & Horvath, S. A general framework for weighted gene co-expression network analysis. *Stat. Appl. Genet. Mol. Biol*. 10.2202/1544-6115.1128 (2005). 10.2202/1544-6115.1128 16646834

[21] Georgouli M (2019). Regional activation of myosin II in cancer cells drives tumor progression via a secretory cross-talk with the immune microenvironment. Cell.

[22] Ayers M (2017). IFN-γ-related mRNA profile predicts clinical response to PD-1 blockade. J. Clin. Invest..

[23] Rooney MS, Shukla SA, Wu CJ, Getz G, Hacohen N (2015). Molecular and genetic properties of tumors associated with local immune cytolytic activity. Cell.

[24] Riaz N (2017). Tumor and microenvironment evolution during immunotherapy with nivolumab. Cell.

[25] Bratman SV (2020). Personalized circulating tumor DNA analysis as a predictive biomarker in solid tumor patients treated with pembrolizumab. Nat. Cancer.

[26] Davis AA, Patel VG (2019). The role of PD-L1 expression as a predictive biomarker: an analysis of all US Food and Drug Administration (FDA) approvals of immune checkpoint inhibitors. J. Immunother. Cancer.

[27] Doroshow DB (2021). PD-L1 as a biomarker of response to immune-checkpoint inhibitors. Nat. Rev. Clin. Oncol..

[28] Dolled-Filhart M (2016). Development of a prototype immunohistochemistry assay to measure programmed death ligand-1 expression in tumor tissue. Arch. Pathol. Lab. Med..

[29] Gröbner SN (2018). The landscape of genomic alterations across childhood cancers. Nature.

[30] McDermott DF (2018). Clinical activity and molecular correlates of response to atezolizumab alone or in combination with bevacizumab versus sunitinib in renal cell carcinoma. Nat. Med..

[31] Lawrence MS (2013). Mutational heterogeneity in cancer and the search for new cancer-associated genes. Nature.

[32] Das A (2022). Genomic predictors of response to PD-1 inhibition in children with germline DNA replication repair deficiency. Nat. Med..

[33] Campbell BB (2017). Comprehensive analysis of hypermutation in human cancer. Cell.

[34] Puig-Saus C (2020). Landscape analysis of neoepitope-specific T-cell responses to immunotherapy. Cancer Res..

[35] Tumeh PC (2014). PD-1 blockade induces responses by inhibiting adaptive immune resistance. Nature.

[36] Roh W (2017). Integrated molecular analysis of tumor biopsies on sequential CTLA-4 and PD-1 blockade reveals markers of response and resistance. Sci. Transl. Med..

[37] Amaria RN (2018). Neoadjuvant immune checkpoint blockade in high-risk resectable melanoma. Nat. Med..

[38] Marijt KA, Doorduijn EM, van Hall T (2019). TEIPP antigens for T-cell based immunotherapy of immune-edited HLA class Ilow cancers. Mol. Immunol..

[39] Doorduijn EM (2016). TAP-independent self-peptides enhance T cell recognition of immune-escaped tumors. J. Clin. Invest..

[40] van Hall T (2006). Selective cytotoxic T-lymphocyte targeting of tumor immune escape variants. Nat. Med..

[41] Doorduijn EM (2018). T cells specific for a TAP-independent self-peptide remain naïve in tumor-bearing mice and are fully exploitable for therapy. OncoImmunology.

[42] Cabrita R (2020). Tertiary lymphoid structures improve immunotherapy and survival in melanoma. Nature.

[43] Helmink BA (2020). B cells and tertiary lymphoid structures promote immunotherapy response. Nature.

[44] Petitprez F (2020). B cells are associated with survival and immunotherapy response in sarcoma. Nature.

[45] Meylan M (2022). Tertiary lymphoid structures generate and propagate anti-tumor antibody-producing plasma cells in renal cell cancer. Immunity.

[46] Zheng L (2021). Pan-cancer single-cell landscape of tumor-infiltrating T cells. Science.

[47] Litchfield K (2021). Meta-analysis of tumor- and T cell-intrinsic mechanisms of sensitization to checkpoint inhibition. Cell.

[48] Chow MT (2019). Intratumoral activity of the CXCR3 chemokine system is required for the efficacy of anti-PD-1 therapy. Immunity.

[49] Lepin EJ (2000). Functional characterization of HLA-F and binding of HLA-F tetramers to ILT2 and ILT4 receptors. Eur. J. Immunol.

[50] Garcia-Beltran WF (2016). Open conformers of HLA-F are high-affinity ligands of the activating NK-cell receptor KIR3DS1. Nat. Immunol..

[51] Dulberger CL (2017). Human leukocyte antigen F presents peptides and regulates immunity through interactions with NK cell receptors. Immunity.

[52] Williamson LM (2021). Clinical response to nivolumab in an INI1-deficient pediatric chordoma correlates with immunogenic recognition of brachyury. NPJ Precis. Oncol..

[53] Blank CU, Haanen JB, Ribas A, Schumacher TN (2016). The ‘cancer immunogram’. Science.

[54] Frampton GM (2013). Development and validation of a clinical cancer genomic profiling test based on massively parallel DNA sequencing. Nat. Biotechnol..

[55] Ross JS (2015). Genomic profiling of advanced-stage, metaplastic breast carcinoma by next-generation sequencing reveals frequent, targetable genomic abnormalities and potential new treatment options. Arch. Pathol. Lab. Med..

[56] Chalmers ZR (2017). Analysis of 100,000 human cancer genomes reveals the landscape of tumor mutational burden. Genome Med..

[57] Dobin A (2013). STAR: ultrafast universal RNA-seq aligner. Bioinformatics.

[58] Li B, Dewey CN (2011). RSEM: accurate transcript quantification from RNA-seq data with or without a reference genome. BMC Bioinform..

[59] Sturm G (2019). Comprehensive evaluation of transcriptome-based cell-type quantification methods for immuno-oncology. Bioinformatics.

[60] Racle J, de Jonge K, Baumgaertner P, Speiser DE, Gfeller D (2017). Simultaneous enumeration of cancer and immune cell types from bulk tumor gene expression data. eLife.

[61] Finotello F (2019). Molecular and pharmacological modulators of the tumor immune contexture revealed by deconvolution of RNA-seq data. Genome Med..

[62] Li B (2016). Comprehensive analyses of tumor immunity: implications for cancer immunotherapy. Genome Biol..

[63] Aran D, Hu Z, Butte AJ (2017). xCell: digitally portraying the tissue cellular heterogeneity landscape. Genome Biol..

[64] Wang TT (2019). High efficiency error suppression for accurate detection of low-frequency variants. Nucleic Acids Res..

[65] Bolotin DA (2015). MiXCR: software for comprehensive adaptive immunity profiling. Nat. Methods.

[66] Chao A (2014). Rarefaction and extrapolation with Hill numbers: a framework for sampling and estimation in species diversity studies. Ecol. Monogr..

[67] Soneson C, Love MI, Robinson MD (2015). Differential analyses for RNA-seq: transcript-level estimates improve gene-level inferences. F1000Res.

[68] Love MI, Huber W, Anders S (2014). Moderated estimation of fold change and dispersion for RNA-seq data with DESeq2. Genome Biol..

[69] Korotkevich, G. et al. Fast gene set enrichment analysis. Preprint at *BioRxiv*10.1101/060012 (2021).

[70] Langfelder P, Horvath S (2007). Eigengene networks for studying the relationships between co-expression modules. BMC Syst. Biol..

[71] Cindy Yang SY (2021). Pan-cancer analysis of longitudinal metastatic tumors reveals genomic alterations and immune landscape dynamics associated with pembrolizumab sensitivity. Nat. Commun..

