# Supplementary material for: Multimodal immunogenomic biomarker analysis of tumors from pediatric patients enrolled to a phase 1-2 study of single-agent atezolizumab
Source: Nat Cancer. 2023 Apr 10;4(4):502–15. doi: 10.1038/s43018-023-00534-x (PMC10132976; doi:10.1038/s43018-023-00534-x)
Supplement: Supplementary file 1 — Reporting Summary [file 43018_2023_534_MOESM1_ESM.pdf]

nature portfolio

Corresponding author(s): Katherine E. Hutchinson
Trevor J. Pugh

Last updated by author(s): Jan 19, 2023

Reporting Summary

Nature Portfolio wishes to improve the reproducibility of the work that we publish. This form provides structure for consistency and transparency in reporting. For further information on Nature Portfolio policies, see our Editorial Policies and the Editorial Policy Checklist.

Statistics

For all statistical analyses, confirm that the following items are present in the figure legend, table legend, main text, or Methods section.

n/a	Confirmed	
☐	☒	The exact sample size (*n*) for each experimental group/condition, given as a discrete number and unit of measurement
☐	☒	A statement on whether measurements were taken from distinct samples or whether the same sample was measured repeatedly
☐	☒	The statistical test(s) used AND whether they are one- or two-sided<br>*Only common tests should be described solely by name; describe more complex techniques in the Methods section.*
☐	☒	A description of all covariates tested
☒	☐	A description of any assumptions or corrections, such as tests of normality and adjustment for multiple comparisons
☐	☒	A full description of the statistical parameters including central tendency (e.g. means) or other basic estimates (e.g. regression coefficient) AND variation (e.g. standard deviation) or associated estimates of uncertainty (e.g. confidence intervals)
☐	☒	For null hypothesis testing, the test statistic (e.g. *F*, *t*, *r*) with confidence intervals, effect sizes, degrees of freedom and *P* value noted<br>*Give P values as exact values whenever suitable.*
☒	☐	For Bayesian analysis, information on the choice of priors and Markov chain Monte Carlo settings
☒	☐	For hierarchical and complex designs, identification of the appropriate level for tests and full reporting of outcomes
☐	☒	Estimates of effect sizes (e.g. Cohen's *d*, Pearson's *r*), indicating how they were calculated

Our web collection on statistics for biologists contains articles on many of the points above.

Software and code

Policy information about availability of computer code

Data collection	Patient clinical data (i.e. from the associated clinical trial) were collected by the clinical trial investigators and their research teams at their respective institutions during the course of the clinical trial via electronic data capture (EDC) (vendor: Medidata) and an interactive voice/web response system (IXRS) (vendor: Bracket, now known as Signant Health).<br>These data were organized using SAS (v9.4), R (v3.5.1), and Microsoft Excel (v16.0 or above).
Data analysis	R v4.0 and Jupyter notebook environment were used for data analysis.<br>RNA-seq data processing: STAR aligner v2.4.2a and RSEM v1.2.29<br>Immune deconvolution analysis: web portal TIMER v2.0, R packages Immunedeconv v2.0.0, CIBERSORT v1.0.4, EPIC v1.1.5, MCPCounter v1.2.0, xCell v1.10, TIMER v0.1, quanTIseq v3.16<br>TCR repertoire analysis: Software MiXCR v2.1.12, R package iNEXT v2.0.20<br>Statistical analysis: R packages survival v3.2-7, survminer v0.4.9<br>Network analysis: R packages GSVA v1.38.2, WGCNA v1.69<br>Differential gene expression analysis: R packages DESeq2 v1.30.0, tximport v1.18.0, fgsea v1.16.0<br>Immunohistochemical (IHC) image analysis: Definiens Architect or Definiens Developer (v2.1.1) and Definiens Tissue Studio (v3.6.1)<br>Figure editing: Adobe Illustrator v24.0.1<br>Custom codes: https://github.com/pughlab/iMATRIX-Atezo_Biomarker

For manuscripts utilizing custom algorithms or software that are central to the research but not yet described in published literature, software must be made available to editors and reviewers. We strongly encourage code deposition in a community repository (e.g. GitHub). See the Nature Portfolio guidelines for submitting code & software for further information.

Data

Policy information about availability of data

All manuscripts must include a data availability statement. This statement should provide the following information, where applicable:

- Accession codes, unique identifiers, or web links for publicly available datasets
- A description of any restrictions on data availability
- For clinical datasets or third party data, please ensure that the statement adheres to our policy

Individual patient-level data, including de-identified clinical metadata, raw RNAseq data, raw TCRseq data, and processed F1 sequencing data are made available to qualified researchers at the European Genome-Phenome Archive under accession number EGAS00001006004. . To request access to such data, researchers can contact devsci-dac-d@gene.com. The data will be released to such requesters with necessary agreements to enforce terms such as security, patient privacy and consent of specified data use, consistent with evolving, applicable data protection laws. For up-to-date details on Roche's Global Policy on the Sharing of Clinical Information and how to request access to related clinical study documents, see here: https://go.roche.com/data_sharing.
Tumor Compendium v11 was downloaded from the Treehouse Childhood Cancer Initiative (https://treehousegenomics.soe.ucsc.edu/public-data/#tumor_v11_polyA). One-hundred and nine (109) publicly available Sequence Read Archive (SRA) files from the CA209-038 study (NCT01621490) were downloaded using the NCBI SRA selector (SRP094781). Clinical data for the CA209-038 study were available on Github (https://github.com/riazn/bms038_analysis/). Gene expression and clinical outcomes data from the INSPIRE trial are available as described before (Yang, S. Y. et al. Pan-cancer analysis of longitudinal metastatic tumors reveals genomic alterations and immune landscape dynamics associated with pembrolizumab sensitivity. Nat Commun 12, 5137 (2021)). Reference human genome (hg38) was downloaded from https://genome.ucsc.edu/

Human research participants

Policy information about studies involving human research participants and Sex and Gender in Research.

Reporting on sex and gender	Participants in the previously reported clinical trial (iMATRIX-atezolizumab, NCT02541604, https://pubmed.ncbi.nlm.nih.gov/31780255/) were enrolled regardless of sex or gender. Of 90 enrolled participants, 49 (54%) were male and 41 (46%) were female. Participant sex was self-reported via electronic data capture (EDC) by the clinical trial site staff. No further analyses by sex were conducted as part of the planned clinical study analyses and consent was not provided to share sex data in a disaggregated (i.e. patient-level) manner.<br><br>For the subsequent exploratory work described in the current manuscript, no analyses were conducted on the basis of sex or gender. The limited response rate and diversity of tumor types enrolled to the clinical study may confound any sex-based analyses and further subgrouping would decrease the power to observe any meaningful biological differences. Related to the intent of the current manuscript, the authors have previously published an analysis of pediatric CNS and neuroblastoma samples which concluded that, independent of tumor type, sex is not associated with the immune microenvironment. See here: https://www.biorxiv.org/content/10.1101/2022.09.20.508719v1
Population characteristics	Details regarding the demographics of the iMATRIX-atezolizumab clinical trial participants can be found in the previously published primary manuscript here: https://pubmed.ncbi.nlm.nih.gov/31780255/<br>Briefly, eighty seven pediatric patients with median age of 14 years were enrolled in the iMATRIX-atezo trial and received at least one dose of atezolizumab. Ten cancer types were included together with one group of rare tumors and were classified into lymphoma and solid tumor groups. Forty-five samples were primary tumors and 21 were metastatic. Other in-depth clinical information are reported previously in the primary clinical manuscript.
Recruitment	Details on participant recruitment to the iMATRIX-atezolizmab clinical trial can be found in the previously published primary manuscript here: https://pubmed.ncbi.nlm.nih.gov/31780255/<br>Briefly, patients younger than 30 years of age with relapsed or refractory solid tumors or lymphoma were enrolled in the iMATRIX-atezo trial. Therefore, our results are biased toward cancers that progressed after multiple lines of therapy and may not be applicable to untreated cancers.
Ethics oversight	As described in the current manuscript and in the primary clinical manuscript, the iMATRIX-atezolizumab study was done in accordance with the principles of the Declaration of Helsinki and Good Clinical Practice, and the protocol was approved by the relevant ethics bodies at each participating site: in Denmark, Rigshospitalet Section of Paediatric Hematology and Oncology Adolescent Medicine by the central De Videnskabsetiske Komitéer Region Hovedstaden; in France, Institut de Cancerologie Gustave Roussy Département de Pediatrie, Institut Curie Département de Pediatrie, and Centre Léon Bérard by the central Comité de Protection des Personnes Ile de France VII; in Germany, Universitätsklinikum Frankfurt Zentrum für Kinder- und Jugendmedizin by the local Ethikkommission des Fachbereichs Medizin der Johann Wolfgang Goethe-Universitat Frankfurt; in Israel, Schneider Children's Medical Center of Israel Department of Pediatric Hematology and Oncology by the local Ethic (Helsinki) Committee at Rabin Medical Center; in Italy, Ospedale Pediatrico Bambino Gesù Dipartimento di Onco-Ematologia Pediatrica e Medicina Trasfusionale by the local Fondazione Instituto de Ricovero e Cura a Carattere Scientifico (IRCCS) Ospedale Pediatrico Bambino Gesù, Azienda Ospedaliero-Universitaria di Padova Oncoematologia Pediatrica by the local Comitato Etico per Sperimentazione Clinica della Provincia di Padova, lstituto Pediatrico di Ricovero e Cura a Carattere Scientifico lstituto Giannina Gaslini by the local Comitato Etico Regione Liguria Sezione 3 in Genova, Azienda Ospedaliera Universitaria Citta della Salute e della Scienza di Torino by the local Comitato Etico Interaziendale in Torino, and Instituto Nazionale dei Tumori by the local Comitato Etico della Fondazione IRCCS in Milan; in the Netherlands, Erasmus University Medical Center (MC) by the central Erasmus MC Medical Ethics Review Committee (METC); in Spain, Hospital lnfantil Universitario Nino Jesus by the central al Comité de Ética de Investigación Clínicas (CEIC) Nino Jesus, Hospital Universitario Vall d'Hebron by the local Unidad de Soporte al CEIC, and Hospital San Joan de Deu Unitat de Recerca Clinica by the local CEIC; in Switzerland, University Children's Hospital Zurich by the central Kantonale Ethikkommission Zurich; in the United

Kingdom, Leeds General Infirmary Leeds Children's Hospital Great George, The Royal Marsden National Health Service (NHS) Foundation Trust, Birmingham Women's and Children's NGS Foundation Trust Birmingham Children's Hospital, Bristol Royal Hospital for Children by the central NRES Committee South Central-Berkshire; and in the United States, each with their corresponding local ethics committee, Arkansas Children's Hospital, Penn State Hershey Children's Hospital, Dana Farber Cancer Institute, MD Anderson Cancer Center Division of Pediatrics, Stanford University School of Medicine Department of Pediatric Hematology and Oncology, University of Texas Health Sciences Center at San Antonio, and Memorial Sloan-Kettering Cancer Center. An independent data monitoring committee monitored patient safety throughout the study.
An independent data monitoring committee monitored patient safety throughout the study.

Note that full information on the approval of the study protocol must also be provided in the manuscript.

Field-specific reporting

Please select the one below that is the best fit for your research. If you are not sure, read the appropriate sections before making your selection.

☒ Life sciences ☐ Behavioural & social sciences ☐ Ecological, evolutionary & environmental sciences

For a reference copy of the document with all sections, see nature.com/documents/nr-reporting-summary-flat.pdf

Life sciences study design

All studies must disclose on these points even when the disclosure is negative.

Sample size	Sixty six unique tumor samples collected pre-therapy from patients enrolled in the iMATRIX-atezo trial (NCT02541604) were included in the study. Because the current study is observational, we did not perform any sample size analysis.
Data exclusions	Eighty three samples were collected from 90 patients enrolled in the iMATRIX-atezo trial. Ten samples were removed due to failed sequencing quality control. Sixty-seven were collected prior to treatment. One patient had two biopsies from lymph nodes, one of which was randomly removed from the analyses herein.
Replication	Due to limited biospecimen availability, immunohistochemical (IHC) staining was performed only once for each tumor sample. Similarly, due to limited biospecimen availability, only one library was prepared for each sequencing experiment. An Illumina PhiX library sequencing run was used to verify reproducibility.
Randomization	The iMATRIX-atezo trial (NCT02541604) was not a randomized study and had no comparator intervention. Patients were enrolled to specific tumor-type cohorts according to their diagnosed tumor type. For the observational study described herein, no randomization was performed.
Blinding	The iMATRIX-atezo trial (NCT02541604) was not a blinded study. Similarly, no blinding was performed for the observational study described herein. Sample processing and sequencing were performed without prior knowledge of clinical parameters. Immunohistochemistry slides were reviewed blindly.

Reporting for specific materials, systems and methods

We require information from authors about some types of materials, experimental systems and methods used in many studies. Here, indicate whether each material, system or method listed is relevant to your study. If you are not sure if a list item applies to your research, read the appropriate section before selecting a response.

Materials & experimental systems

n/a	Involved in the study	
☐	☒	Antibodies
☒	☐	Eukaryotic cell lines
☒	☐	Palaeontology and archaeology
☒	☐	Animals and other organisms
☐	☒	Clinical data
☒	☐	Dual use research of concern

Methods

n/a	Involved in the study	
☒	☐	ChIP-seq
☒	☐	Flow cytometry
☒	☐	MRI-based neuroimaging

Antibodies

Antibodies used

Immunohistochemistry (IHC) was performed retrospectively in batch during the course of the iMATRIX-atezolizumab clinical trial on patient tumor tissue samples at Ventana for PD-L1 and at HistoGeneX (now known as CellCarta) for CD3, CD8, and CD20.
VENTANA PD-L1 (SP142) Antibody Assay: Supplier, Roche-Ventana; Catalog ID, Ventana 740-4859; clone SP142 (rabbit monoclonal antibody); lots E04424R and G00171; supplied in ready-to-use dispensers (i.e. no dilution)

CONFIRM anti-CD3 primary antibody: Supplier, Roche-Ventana; Catalog ID, Ventana 790-4341; clone 2GV6 (rabbit monoclonal primary antibody); multiple lots; supplied in ready-to-use dispensers (i.e. no dilution)

anti-CD8 primary antibody: Supplier, DAKO; Catalog ID, DAKO M7103; clone C8/144B (mouse monoclonal primary antibody); multiple lots; primary antibody supplied at 157 µg/mL and diluted 1:75 in Ventana Benchmark Diluent (Ventana 251-018) to final concentration of 2.1 µg/mL

CONFIRM anti-CD20 primary antibody: Supplier, Roche-Ventana; Catalog ID, Ventana 760-2531; clone L26 (mouse monoclonal antibody); multiple lots; supplied in ready-to-use dispensers (i.e. no dilution)

Validation

All antibodies were used according to the manufacturer's instructions and validated by testing vendors for exploratory purposes, with monitoring for accuracy and precision (i.e. compared against isotype or no staining control samples).

Details for each antibody are available at:
VENTANA PD-L1 (SP142) Antibody Assay:
via Roche Diagnostics eLab Doc site (https://pim-eservices.roche.com/eLD/web/us/en/home) to
https://pim-eservices.roche.com/eLD/api/downloads/2456f3c8-2a9a-ea11-fc90-005056a71a5d?countryIsoCode=us

CONFIRM anti-CD3 primary antibody:
via Roche Diagnostics eLab Doc site (https://pim-eservices.roche.com/eLD/web/us/en/home) to
https://pim-eservices.roche.com/eLD/api/downloads/49729da6-7333-ea11-fa90-005056a772fd?countryIsoCode=us

anti-CD8 primary antibody:
https://www.agilent.com/en/product/immunohistochemistry/antibodies-controls/primary-antibodies/cd8-(dako-omnis)-76236

CONFIRM anti-CD20 primary antibody:
via Roche Diagnostics eLab Doc site (https://pim-eservices.roche.com/eLD/web/us/en/home) to
https://pim-eservices.roche.com/eLD/api/downloads/50d4b69b-6833-ea11-fa90-005056a772fd?countryIsoCode=us

Clinical data

Policy information about clinical studies

All manuscripts should comply with the ICMJE guidelines for publication of clinical research and a completed CONSORT checklist must be included with all submissions.

Clinical trial registration

The iMATRIX-atezolizumab clinical trial from which samples were analyzed for the exploratory study herein is registered with clinicaltrials.gov, number NCT02541604, linked here: https://clinicaltrials.gov/ct2/show/NCT02541604

The primary clinical manuscript is linked here: https://pubmed.ncbi.nlm.nih.gov/31780255/

Study protocol

The study protocol can be accessed as part of the supplement to the primary clinical manuscript here: https://pubmed.ncbi.nlm.nih.gov/31780255/

Data collection

The iMATRIX-atezo clinical trial was designed by the sponsor/funder (F Hoffmann-La Roche) and the clinical trial investigators. The trial sponsors, F Hoffmann-La Roche and Genentech, were involved in the administration and conduct of study procedures, coordination of data collection, data analysis, and data interpretation. During the trial enrollment period (November 2015 through April 2018), individual patient data were collected by the clinical trial investigators and their research teams at their respective institutions, and were interpreted by the sponsor/funder and all authors of the primary clinical manuscript (https://pubmed.ncbi.nlm.nih.gov/31780255/).

Outcomes

The primary endpoints of the iMATRIX-atezo clinical trial were the safety (assessed by incidences of adverse events) and pharmacokinetics (assessed by serum concentrations) of atezolizumab.

Secondary endpoints were preliminary antitumor activity of atezolizumab (proportion of patients achieving an objective response, clinical benefit, and progression-free survival, including duration of objective response and overall survival) and immunogenicity of atezolizumab (frequency of treatment-emergent anti-drug antibodies relative to anti-drug antibody prevalence at baseline).

Correlation between response to atezolizumab and PD-L1 tumor expression was a prespecified exploratory endpoint.

See also the primary clinical manuscript (link in field above).
